# Autism spectrum disorder causes, mechanisms, and treatments: focus on neuronal synapses

**DOI:** 10.3389/fnmol.2013.00019

**Published:** 2013-08-05

**Authors:** Hyejung Won, Won Mah, Eunjoon Kim

**Affiliations:** ^1^Department of Biological Sciences, Korea Advanced Institute of Science and TechnologyDaejeon, South Korea; ^2^Center for Synaptic Brain Dysfunctions, Institute for Basic ScienceDaejeon, South Korea

**Keywords:** autism spectrum disorder, therapeutics, genetics, animal model, synapse, synaptopathy

## Abstract

Autism spectrum disorder (ASD) is a group of developmental disabilities characterized by impairments in social interaction and communication and restricted and repetitive interests/behaviors. Advances in human genomics have identified a large number of genetic variations associated with ASD. These associations are being rapidly verified by a growing number of studies using a variety of approaches, including mouse genetics. These studies have also identified key mechanisms underlying the pathogenesis of ASD, many of which involve synaptic dysfunctions, and have investigated novel, mechanism-based therapeutic strategies. This review will try to integrate these three key aspects of ASD research: human genetics, animal models, and potential treatments. Continued efforts in this direction should ultimately reveal core mechanisms that account for a larger fraction of ASD cases and identify neural mechanisms associated with specific ASD symptoms, providing important clues to efficient ASD treatment.

## Introduction to autism spectrum disorder

Autism spectrum disorder (ASD) is a group of developmental disabilities characterized by abnormal social interaction and communication, and stereotyped behaviors with restricted interest. Autism was first reported by Kanner (1943) with a clinical description of 11 children showing “extreme aloneness from the very beginning of life, not responding to anything that comes to them from the outside world.” He proposed the behavioral combination of autism, obsessiveness, stereotypy, and echolalia as childhood schizophrenia. However, until the 1980s, ASD was not accepted as an individual developmental disorder with a biological origin. In the early 1980s, studies demonstrated the high heritability of ASD and its association with other genetic syndromes (Gillberg and Wahlstrom, 1985; Wahlstrom et al., 1986), providing compelling evidence for a genetic etiology of ASD and fueling the conceptualization of autism as a distinct neurodevelopmental disorder. From the definition of “childhood or early-onset schizophrenia” put forward by Kanner, autism was renamed “infantile autism” in 1980, “autism disorder” in 1987 and, more recently, “autism” or the umbrella term “ASD”.

### Diagnosis

Currently, ASD is included in the diagnostic category of a neurodevelopmental disorders in the Diagnostic and Statistical Manual of Mental Disorders V (Grzadzinski et al., 2013). The diagnosis of autism is mainly based on the presence of two major aforementioned symptoms: social-communication deficits, and restricted and repetitive interests/behaviors (Grzadzinski et al., 2013). These symptoms must be shown from early childhood of individuals with ASD. But autism is also associated with various comorbidities, including sensory and motor abnormalities, sleep disturbance, epilepsy, attention deficit/hyperactivity disorder (ADHD)-like hyperactivity, intellectual disability, and mood disorders such as anxiety and aggression (Goldstein and Schwebach, 2004; Simonoff et al., 2008; Geschwind, 2009). Some monogenic syndromes including fragile X syndrome and Rett syndrome also have autistic features, while we should be cautious to directly interpret the disorders as autism since the major symptoms for these syndromes are intellectual disabilities.

### Prevalence

An early study conducted in the UK in 1966 reported a prevalence rate of autism of 4.5 in 10,000 children (Lotter, 1966). The estimated prevalence increased to 19 in 10,000 American children in 1992 and rose steeply to 1 in 150 in 2002 (Autism et al., 2007) and 1 in 110 in 2006 (Autism et al., 2009) (see also data from the US Centers for Disease Control and Prevention [CDC]). The currently accepted prevalence of ASD, based on consistent reports of ASD prevalence by multiple sources in different populations, is ~1% worldwide, placing this disorder as one of the most common pervasive developmental disorders and elevating public concerns.

### Genetics

On the basis of numerous studies that have been undertaken to elucidate the pathogenic mechanisms underlying ASD, it is widely accepted that ASD is a disorder with strong genetic components. In support of this notion, the concordance rates for autism reach up to 90% in monozygotic twins and 10% in dizygotic twins (Rutter, 2000; Folstein and Rosen-Sheidley, 2001; Veenstra-Vanderweele et al., 2003).

However, autism is an etiologically heterogeneous disorder in that no single genetic mutation accounts for more than 1–2% of ASD cases (Abrahams and Geschwind, 2008). Thus far, linkage and candidate-gene analyses, genome-wide association studies (GWAS), and assessments of chromosomal variations have uncovered a wide range of genes with predisposing mutations and polymorphisms associated with ASD (International Molecular Genetic Study of Autism, 1998, 2001; Abrahams and Geschwind, 2008; Glessner et al., 2009; Ma et al., 2009; Wang et al., 2009; Weiss et al., 2009; Anney et al., 2010; Pinto et al., 2010; Devlin and Scherer, 2012; Moreno-De-Luca et al., 2013) (see Tables 1, 2 for examples). Moreover, recent advancements in exome sequencing and next-generation sequencing have enabled the discovery of an overwhelming number of *de novo* mutations that confer a risk for ASD (Iossifov et al., 2012; Neale et al., 2012; O'Roak et al., 2012a,b; Sanders et al., 2012). These mutations include rare mutations or copy number variations in synaptic proteins such as Shanks/ProSAPs (Durand et al., 2007; Berkel et al., 2010; Sato et al., 2012) and neuroligins (Jamain et al., 2003).

**Table 1:** **Examples of ASD-associated chromosomal loci and candidate genes from GWAS**.

**Chromosomal loci**	**Candidate genes**	**Sample size**	**Design**	**Population**	**References**
4p, 7q, 16p	*GPR37, PTPRZ1, EPHB6, PTN, CASP2, GRM8, EAG* in 7q region	87 affected sib pairs and 12 non-sib affected relative pairs	Family	99 Caucasian families (66 from the UK, 11 from Germany, 10 from the Netherlands, 5 from USA, 5 from France, 2 from Denmark)	International Molecular Genetic Study of Autism, 1998
2q, 4q, 5p, 6q, 7q, 10q, 15q11-q15, 16p, 18q, 19p, Xp	*GABRB3 in* 15q11-q15 region, *MACS GRIK6, GPR6* in 6q region	51 families including at least two siblings or half-siblings affected by autism	Family	51 Caucasian families (18 from Sweden, 15 from France, 6 from Norway, 5 from the USA, 3 from Italy, 2 from Austria and 2 from Belgium)	Philippe et al., 1999
5p14.1	*CDH9, CDH10* in 5p14.1 region	943 families	Family	Autism Genetic Resource Exchange (AGRE)	Wang et al., 2009
5p14.1	*CDH9* and *CDH10* in 5p14.1 region	487 families	Family	487 Caucasian families (80 multiplex families, 407 singleton familes)	Ma et al., 2009
5p15, 6q27, 20p13	*TAS2R1* and *SEMA5A* in 5p15 region	1031 multiplex families	Family	AGRE and US National Institute for Mental Health (NIMH)	Weiss et al., 2009
20p12.1	*MACROD2* in 20p12.1	1558 families	Family	Autism Genome Project (AGP)	Anney et al., 2010

**Table 2:** **Examples of ASD-associated human genetic variations**.

**Genes**	**CNV/SNV**	**Sample size**	**Design**	**Population**	**References**
MET	rs1858830	743 autism families, 702 unrelated autism patients/189 unrelated controls	Case/control, family	Italian and American population	Campbell et al., 2007
WNT2	linkage disequilibrium in Wnt 3′UTR, R299W, L5R	75 autism-affected sibling pair families (ASP)	Trio	Families recruited from three regions of the United States (Midwest, New England, and mid-Atlantic states)	Wassink et al., 2001
	rs3779547, rs4727847, rs3729629	170 autism patients/214 normal controls	Case/control	Japanese population	Marui et al., 2010
RELN	5′ UTR polymorphic GGC repeats	371 families	Family	Caucasian	Skaar et al., 2005
		172 autism trios, 95 unrelated autism patients/186 unrelated controls	Case/control, trio	Italian and American population	Persico et al., 2001
EN2/ ENGRAILED-2	rs1861972, rs1861973	518 families	Family	AGRE and National Institutes of Mental Health (NIMH)	Benayed et al., 2005; Gharani et al., 2004
HOXA1	A218G	57 probands, 166 relatives	Probands/relatives	Not identified	Ingram et al., 2000b
CHD8	*de novo* frameshift, nonsense mutations	209 trios	Trio	Simons Simplex Collection (SSC)	O'Roak et al., 2012a
GRIK2 (GluR6)	M867I	59 ASP, 107 trios	Family	Families recruited from 7 countries (Austria, Belgium, France, Italy, Norway, Sweden, US)	Jamain et al., 2002
GRM8	R859C, R1085Q, R1100Q, intrachromosomal segmental duplication	196 multiplex families	Family	AGRE	Serajee et al., 2003
GRIN2A (GluN2A)	rs1014531	219 sibling pairs, 32 families with extended relative pairs	Family	International Molecular Genetics Study of Autism Consortium (IMGSAC)	Barnby et al., 2005
GRIN2B (GluN2B)	*de novo* protein truncating and splicing mutations	209 trios	Trio	Simons Simplex Collection (SSC)	O'Roak et al., 2012a
GABRB3	Linkage disequilibrium	138 families, mainly trio	Family	104 Caucasian, 6 African American, 13 Asian American, 5 Hispanic	Cook et al., 1998
	Transmission disequilibrium	70 families	Trio	AGRE, Seaver Autism Research Center (SARC)	Buxbaum et al., 2002
5-HTT	Transmission disequilibrium	86 trios	Trio	68 Caucasian, 5 African American, 3 Hispanic American, 10 Asian American	Cook et al., 1997
TPH2	rs4341581, rs11179000	88 autistic subjects, 95 unrelated controls	Case/control	people from Utah	Coon et al., 2005
NRXN1	rs1363036, rs930752, hemizygous CNV deletion of coding exons of NRXN1	1491 families	Family	Autism Genome Project (AGP) Consortium	Autism Genome Project et al., 2007
	L18Q, L748I, rs1045874	57 ASD subjects, 27 OCD subjects, 30 Tourette syndrome subjects	Case/control	Developmental Genome Anatomy Project (DGAP)	Kim et al., 2008
NLGN3	R451C	36 sibling pairs, 122 trios	Trio	not identified	Jamain et al., 2003
NLGN4	Frameshift mutation by 1bp insertion (1186InsT)				
SHANK1	*de novo* deletion	1614 ASD subjects, 15000 controls	Case/control	1158 Canadian, 456 European	Sato et al., 2012
SHANK2	CNV deletion for premature stop, R26W, P208S, R462X, T1127M, A1350T, L1008_P1009dup	396 ASD cases, 184 MR cases, 659 controls	Case/control	Canadian for ASD, German for MR	Berkel et al., 2010
	R443C, R598L, V717F, A729T, E1162K, G1170R, V1376I, D1535N, L1722P	851 ASD cases, 1090 controls	Case/control	Paris Autism Research International Sibpair (PARIS)	Leblond et al., 2012
SHANK3	R12C, A198G, R300C, G1011V, R1066L, R1231H, *de novo* frameshift mutation, *de novo* truncating mutation	227 families	Family	PARIS	Durand et al., 2007
CNTNAP2	rs2710102	476 trios	Trio	AGRE	Alarcon et al., 2008
	rs779475	72 families	Family	NIMH	Arking et al., 2008
	Nonsynonymous variants, I869T	635 patients, 942 controls	Case/control	587 white, 24 white-Hispanic, 7 unknown, 6 Asian, 6 more than one race, 3 African-American, 1 Native Hawaiian, 1 more than one race-Hispanic	Bakkaloglu et al., 2008
	rs17236239	184 families	Family	Specific Language Impairment Consortium (SLIC)	Vernes et al., 2008
ILRAPL1	Frameshift	142 ASD case, 189 controls	Case/control	85 French Canadians, 47 European Caucasians, 10 non-Caucasians	Piton et al., 2008
OPHN1	Frameshift				Piton et al., 2011
SYNGAP1	CNV deletion	996 ASD cases, 1287 controls	Case/control	European	Pinto et al., 2010
TM4SF2	Nonsynonymous variants, P172H	142 ASD case, 189 controls	Case/control	85 French Canadians, 47 European Caucasians, 10 non-Caucasians	Piton et al., 2011

However, how these mutations lead to ASD phenotypes is poorly understood. In addition, many ASD-related genes are also associated with other neuropsychiatric disorders. For example, *IL1RAPL1* and *OPHN1* are associated with X chromosome-linked intellectual disability (Billuart et al., 1998a; Carrie et al., 1999). Additional examples include schizophrenia for *RELN*, *GluR6*, *GRIN2A*, *GRIN2B*, and *CNTNAP2* (Bah et al., 2004; Friedman et al., 2008; Shifman et al., 2008; Demontis et al., 2011), childhood absence epilepsy for *GABRB3* (Feucht et al., 1999), ADHD and depression for *5-HTT* (Manor et al., 2001; Caspi et al., 2003), and major depression for *TPH2* (Zill et al., 2004). Dissecting the neural mechanisms underlying diverse symptoms/disorders caused by single genetic defects is one of the key directions for neuropsychiatric research.

### Animal models for ASD

Animal models of human diseases need to satisfy three major criteria; face validity, construct validity, and predictive validity. Animal models for ASD should display behavioral abnormalities, including impaired sociability, impaired social communication, and repetitive and restricted behaviors (face validity). These models should share analogous genetic or anatomical impairments with humans (construct validity), and show similar responses to the medications used to treat ASD in humans (predictive validity).

Dedicated efforts of many behavioral neuroscientists including Jacqueline Crawley led to the establishment of several well-known assays for rat/mouse models of ASD (Silverman et al., 2010b). Examples include 3-chambered test to assess sociability and social novelty recognition of rodents, ultrasonic vocalization (USV) test to measure the communication patterns of rodents, *T*-maze test for restricted interests, and home cage behavior or marble burying assay for repetitive behaviors. Through these assays, many genetic and non-genetic animal models of ASD have been characterized and used to identify the etiology of ASD and develop novel treatments (see Tables 3–6 for four different groups of ASD models).

**Table 3:** **ASD models with chromosomal abnormality**.

**Mouse**	**Molecular function**	**Phenotype**				**Suggested mechanism**	**References**
		**Social interaction**	**Social communication**	**Repetitive behavior**	**Other phenotypes**		
15q11-13 duplication	Ube3a, Gabr	Impaired	Reduced calls	Behavioral inflexibility	NA	Altered serotonergic signaling	Nakatani et al., 2009
16p11.2 CNV	Kif22, Mapk3	NA	NA	Climbing deficits	Altered diurnal rhythm	Hypothalamic Deficits	Horev et al., 2011
22q11.2 microdeletion	Dgcr2, Comt, Dgcr8	NA	NA	NA	Hyperactivity, Sensorygating deficits	Altered microRNA biogenesis	Stark et al., 2008

**Table 4:** **Syndromic ASD models**.

**Mouse**	**Molecular function**	**Phenotype**				**Suggested mechanism**	**Potential therapeutics**	**Syndrome**	**References**
		**Social interaction**	**Social communication**	**Repetitive behavior**	**Other phenotypes**				
MeCP2 KO	Transcriptional regulator	Impaired (enhanced interaction)	Increased scent marking	Hindlimb clasping	Respiratory problem, Lethality	Decreased GABAergic transmission	BDNF, IGF-1	Rett syndrome	Shahbazian et al., 2002; Moretti et al., 2005
FMR1 KO	Translational repressor	Impaired	NA	Hand flapping	Learning deficits, Anxiety	mGluR hyperfunction	MPEP	FXS	Bernardet and Crusio, 2006
TSC1 HT, TSC1^Cb^ KO	Tumor suppressor	Impaired	Increased calls	Grooming, Behavioral inflexibility	Ataxia	Cerebellar deficits	Rapamycin	Tuberous sclerosis	Auerbach et al., 2011; Tsai et al., 2012
TSC2 HT		Impaired	Increased calls	Increased marble burying	Learning deficits	mGluR hypofunction	Rapamycin, CDPPB		Ehninger et al., 2008
NF1 HT	Tumor suppressor	Impaired	NA	NA	Learning deficits	Ras signaling hyperactivation	NA	Neurofibromatosis	Costa et al., 2001, 2002
PTEN KO	Tumor suppressor	Impaired	NA	NA	Learning deficits	PI3K pathway hyperactivation, Macrocephaly	NA	Cowden/Lhermitte-Duclos syndrome	Kwon et al., 2006
CNTNAP2 KO	Neuron-glia interaction, K^+^ channel cluster	Impaired	Reduced calls	Grooming	Seizure	Reduced number of interneurons, Abnormal neuronal migration	Risperidone	CDFE	Penagarikano et al., 2011
Scn1a KO	Na^+^ channel	Impaired	NA	Grooming	Seizure	Decreased GABAergic transmission	Clonazepam	Dravet's syndrome	Han et al., 2012

**Table 5:** **Synaptopathy ASD models**.

**Mouse**	**Molecular function**	**Phenotype**				**Suggested mechanism**	**Potential therapeutics**	**References**
		**Social interaction**	**Social communication**	**Repetitive behavior**	**Other phenotypes**			
NLGN1 KO	Synaptic adhesion molecule	Minimal impairment	NA	Grooming	Learning deficits	NMDAR hypofunction	D-cycloserine	Blundell et al., 2010
NLGN2 Tg		Impaired	NA	Jumping	Seizure (EEG)	Increased GABAergic transmission	NA	Hines et al., 2008
NLGN3 R451C KI		Impaired	Increased calls	NA	Enhanced learning	Increased GABAergic transmission	NA	Tabuchi et al., 2007
NLGN3 KO		Impaired	Reduced calls	Normal behavioral flexibility	Hyperactivity	Decreased brain volume, Cerebellar deficit	NA	Radyushkin et al., 2009; Baudouin et al., 2012;
NLGN4 KO		Impaired, Less aggression	Reduced calls	Normal	NA	Decreased brain volume	NA	Jamain et al., 2008
Shank1 KO	Synaptic scaffolding protein	Not impaired	Not impaired	Not impaired	Anxiety, Motor deficits	Impaired glutamatergic transmission	NA	Hung et al., 2008; Silverman et al., 2011
Shank2^exon7^ KO		Impaired	Reduced calls, Pattern change	Grooming	Hyperactivity, Anxiety	NMDAR hyperfunction	NA	Schmeisser et al., 2012
Shank2^exon6-7^ KO		Impaired	Reduced calls	Jumping	Hyperactivity, Anxiety	NMDAR hypofunction	CDPPB, DCS	Won et al., 2012
Shank3 HT		Impaired	Reduced calls	NA	NA	Impaired glutamatergic transmission	Ampakine, IGF-1	Bozdagi et al., 2010
Shank3B KO		Impaired	NA	Grooming	Anxiety	Striatal dysfunction	NA	Peca et al., 2011
Shank3^exon4-9^ KO		Impaired	Pattern change	Grooming	Learning deficits	Impaired glutamatergic transmission	NA	Wang et al., 2011
Cadps2 KO	Ca^2+^-dependent secretion activating protein	Impaired, Maternal neglect	NA	NA	Hyperactivity, Anxiety	Decreased density of PV interneuron, Reduced BDNF release	BDNF	Sadakata et al., 2007
Syngap1 HT	GTPase-activating protein for Ras small GTPase	Normal social interaction, Impaired social recognition, Propensity toward social isolation	NA	Stereotypy (repeated single beam break within 1 s)	Learning deficits, Hyperactivity, Anxiolytics, Abnormal sensory-motor gating	Premature spine maturation and hyperexcitability	NA	Guo et al., 2009; Clement et al., 2012

**Table 6:** **Non-synaptopathy ASD models**.

**Mouse**	**Molecular function**	**Phenotype**				**Suggested mechanism**	**Potential therapeutics**	**References**
		**Social interaction**	**Social communication**	**Repetitive behavior**	**Other phenotypes**			
Dvl1 KO	Wnt signaling pathway	Impaired	Not impaired	NA	Sensory gating deficits	Impaired Wnt signaling	NA	Lijam et al., 1997; Long et al., 2004
Oxtr KO	Oxytocin receptor	Impaired, Less aggression	Reduced calls	Not impaired	NA	Impaired oxytocin signaling	Oxytocin	Takayanagi et al., 2005; Crawley et al., 2007
Engrailed-2 KO	Homeodomain transcription factor	Impaired	Not impaired	Not impaired	Learning deficits	Cerebellar deficits	NA	Brielmaier et al., 2012
Reeler	Extracellular matrix glycoprotein	Impaired, Increased social dominance	Reduced calls	NA	Learning deficits, Ataxia	Lissencephaly	Neonatal estrogen	Salinger et al., 2003; Lalonde et al., 2004
4E-BP2 KO	Translational control, mTOR downstream signal	Impaired	Increased call number and duration	Grooming, Increased marble burying	NA	Increased NLGNs translation, Increased excitation	4EGI-1 sh-NLGNs	Gkogkas et al., 2013
eIF4E O/E		Impaired	NA	Grooming, Increased marble burying	Impaired reversal learning, Impaired fear extinction	E/I imbalance in PFC, Increased LTP in striatum and hippocampus	4EGI-1	Santini et al., 2013

Although animal models are useful for exploring ASD mechanisms and testing novel interventions, we should be cautious in interpreting the results from animal models of ASD because what we are observing in animals are behavioral features that look similar to some of the ASD symptoms in humans. This notion partly stems from the fact that the brains of humans and rodents are fundamentally different. For instance, there are small but significant differences in gene expression patterns in the cerebral cortex in different species (Zeng et al., 2012), suggesting that the same cell types in different species may have different functions. Moreover, the size, structural complexity, and neural connectivity of the human brain are much greater than those in rodent brains. These functional and anatomical differences between species may create difficulties in translating the ASD-related mechanisms identified in model organisms into human applications. However, some fundamental aspects of the neural mechanisms identified in animal models such as alterations in synaptic transmission, excitation-inhibition balance, and neuronal excitability might be conserved across species and translatable. In addition, given that stem cell technologies are rapidly improving, it is becoming easier for the changes observed in rodent neurons to be compared with those in human neurons derived from individuals with neuropsychiatric disorders (Brennand et al., 2011).

## Potential mechanisms underlying ASD

Mechanisms underlying autism have been extensively studied using various approaches. Neuroanatomical studies have reported macrocephaly and abnormal neuronal connectivity in autistic individuals, while genetics studies using mouse models have implicated a variety of neuronal proteins in the development of ASD. More recently, defects in a number of synaptic proteins have been suggested to cause ASD via alterations in synaptic structure/function and neural circuits, suggesting that “synaptopathy” is an important component of ASD.

### Neuroanatomical abnormalities

A change frequently observed in the brains of individuals with ASD is the overgrowth of the brain termed macrocephaly, which is observed in ~20% of autistic children (Bolton et al., 2001; Courchesne, 2002; Courchesne et al., 2003, 2007; Fombonne et al., 1999; Hazlett et al., 2005). Aberrations in cytoarchitectural organization in autistic brains are observed during early brain development in regions including the frontal lobe, parieto-temporal lobe, cerebellum, and subcortical limbic structures (Fombonne et al., 1999; Bolton et al., 2001; Courchesne, 2002; Courchesne et al., 2003, 2007; Hazlett et al., 2005).

The cerebellum is a strong candidate for anatomic abnormalities in autism (Courchesne, 1997, 2002). Magnetic resonance imaging (MRI) studies have found hypoplasia of the cerebellar vermis and hemispheres, and autopsy studies have reported a reduction in the number of cerebellar Purkinje cells. In line with these anatomical changes, cerebellar activation is significantly reduced during selective attention tasks (Allen and Courchesne, 2003), whereas it is enhanced during a simple motor task (Allen et al., 2004). Although the putative role of the cerebellum in ASD has been restricted to sensory and motor dysfunctions, it is becoming increasingly clear that the cerebellum is associated with the core symptoms of autism.

In support of this notion, selective deletion of *Tsc1* (tuberous sclerosis 1) in cerebellar Purkinje cells is sufficient to cause all core autism-like behaviors in mice in association with reduced excitability in Purkinje cells (see also Table 4 for summary of syndromic ASD models) (Tsai et al., 2012). In addition, mice lacking the neuroligin-3 gene (*Nlgn3*^−/−^ mice), another autism model with an *Nlgn3* deletion identified in autistic patients, show occluded metabotropic glutamatergic receptor (mGluR)-dependent long-term depression (LTD) at synapses between parallel fibers and Purkinje cells in association with motor coordination deficits (see also Table 5 for summary of synaptopathy ASD models) (Baudouin et al., 2012). Both synaptic and behavioral perturbations are rescued by Purkinje cell-specific re-expression of Nlgn-3 in juvenile mice, suggesting the interesting possibility that altered neural circuits can be corrected after completion of development.

The cerebral cortex is another brain region frequently affected in ASD. Abnormal enlargement or hyperplasia of the cerebral cortex has been reported in MRI studies on young children with ASD (Sparks et al., 2002; Herbert et al., 2003). Because frontal and temporal lobes are important for higher brain functions including social functioning and language development, these anatomical anomalies are likely to underlie the pathophysiology of autism.

The amygdala and hippocampus are subcortical brain regions associated with ASD (Aylward et al., 1999; Schumann et al., 2004; Schumann and Amaral, 2006). Some studies have reported that the autistic amygdala exhibits early enlargement, whereas others have reported a reduction in neuron numbers and amygdala volume. Increases and decreases in the volume of hippocampus are also associated with ASD.

Aberrant connectivity is an emerging theory to account for anatomical abnormalities in autism. Neuroimaging techniques, such as diffusion tensor imaging (DTI) and functional MRI (fMRI), have suggested that ASD involves abrogation of white matter tracts in brain regions associated with social cognition, such as the prefrontal cortex, anterior cingulate cortex, and superior temporal regions (Barnea-Goraly et al., 2004; Minshew and Williams, 2007). Alterations in connectivity across diverse brain regions associated with language, working memory, and social cognition have also been linked to autism. In general, it appears that autism subjects display local over-connectivity and long-range or inter-regional under-connectivity (Herbert et al., 2003, 2004; Baron-Cohen and Belmonte, 2005; Herbert, 2005; Just et al., 2007).

Potential ASD-related neural circuitries have also been proposed based on animal studies. *Shank3b*^−/−^ mice, which exhibit autistic-like behaviors, have striatal dysfunctions (Table 5) (Peca et al., 2011). In addition, a shift in the balance between excitation and inhibition (E-I balance) toward excitation in the mouse medial prefrontal cortex (mPFC) induced by optogenetic stimulation causes sociability impairments (Yizhar et al., 2011). These results suggest that the striatum and mPFC are components of ASD-related neural circuits.

Although various neuroanatomical defects are observed in autistic brains, a direct linkage between neuroanatomical anomalies and behavioral symptoms of ASD remains to be elucidated. Uncovering the detailed circuitries underlying autistic behaviors would help us understand higher cognitive functions, such as language and sociability.

### Extracellular factors

**Figure 1:**
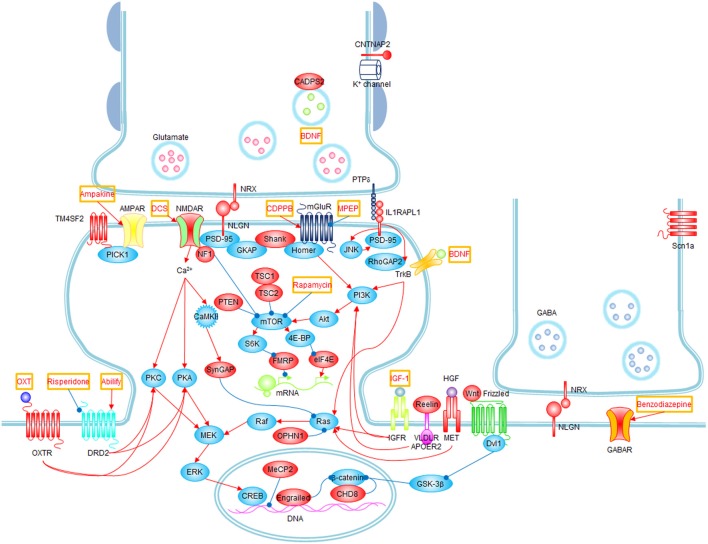
**Signaling pathways and possible treatments associated with ASD**. Molecules whose mutations or polymorphisms are associated with ASD are indicated in red. Stimulations and inhibitions are indicated by red and blue arrows, respectively. Possible treatments and their target molecules are indicated by red texts in orange boxes. SynGAP1, which directly interacts with PSD-95, could not be placed next to PSD-95 for simplicity.

It has been found that growth factors and neurotrophic factors are associated with ASD. Genetic and protein expression studies have shown that MET, a transmembrane receptor for hepatocyte growth factor (HGF) with tyrosine kinase activity, is associated with ASD. Genetic variations including rs1858830 in the promoter region that abrogate MET transcription are associated with ASD in Italian and American families and case/control studies, and the levels of MET mRNA and protein are reduced in the cortex of autistic patients (Campbell et al., 2007, 2006). However, this association between rs1858830 and ASD failed to replicate in another study (Sousa et al., 2009). By binding to MET, HGF acts as a neurotrophic factor for neurons to influence neurite outgrowth and dendritic morphology (Figure 1) (Powell et al., 2001, 2003; Sun et al., 2002; Gutierrez et al., 2004), implicating abnormal neuronal structures in ASD pathology.

WNT2 is a secreted growth factor that has been linked to ASD. Acting through the canonical Wnt pathway, WNT2 triggers a signal transduction cascade mediated by Dishevelled (Dvl1). WNT2 is a critical regulator of multiple biological functions, including embryonic development, cellular differentiation, and cell-polarity generation. It also regulates neuronal migration, axon guidance, and dendrite branching (Figure 1) (Logan and Nusse, 2004). Multiple lines of evidence have implicated the *WNT2* locus in ASD: the *WNT2* gene is located at the autism-susceptibility chromosomal locus 7q31 (Vincent et al., 2000; Warburton et al., 2000), and single nucleotide polymorphisms (SNP; rs3779547, rs4727847, and rs3729629, in a case/control study in a Japanese population) and several *WNT2* locus variants (R299W and L5R, in autism-affected sibling pair [ASP] and trio families) are associated with autism (Wassink et al., 2001; Marui et al., 2010), although a subsequent study in Han Chinese trios failed to replicate the SNP association with ASD (Chien et al., 2011). While the majority of *Wnt2*^−/−^ mice are lethal (Goss et al., 2009), null mutants of *Dvl1* show deficits in nest building and home-cage huddling (see also Table 6 for summary of non-synaptopathy ASD models) (Lijam et al., 1997; Long et al., 2004). Moreover, the Wnt signaling pathway is associated with and is regulated by chromodomain-helicase-DNA-binding protein 8 (CHD8; Figure 1), *de novo* mutations of which are repeatedly detected in autistic patients (Neale et al., 2012; O'Roak et al., 2012b; Sanders et al., 2012).

Brain-derived neurotrophic factor (BDNF) is associated with ASD. BDNF is a member of the neurotrophin family of growth factors that supports neurogenesis, axodendritic growth, neuronal/synaptic differentiation, and brain dysfunctions (Figure 1) (Huang and Reichardt, 2001; Martinowich et al., 2007). Elevated levels of BDNF were reproducibly found in the sera of Japanese and American autistic individuals (Connolly et al., 2006; Miyazaki et al., 2004). Another clue comes from calcium-dependent secretion activator 2 (CADPS2), a calcium binding protein in the presynaptic nerve terminal that interacts with and regulates exocytosis of BDNF-containing dense-core vesicles (Figure 1) (Cisternas et al., 2003). *CADPS2*, located at the autism-susceptibility locus on chromosome 7q31, is abnormally spliced in autism patients, and *Cadps2*^−/−^ mice exhibit social interaction deficits, including maternal neglect (Table 5) (Sadakata et al., 2007). Hence, although it is unclear how BDNF contributes to autism pathogenesis, evidence for its role in ASD is becoming clear.

Reelin is also involved in autism. Reelin is a large secreted extracellular matrix glycoprotein that acts as a serine protease for the extracellular matrix, a function that is essential for neuronal migration, cortical patterning, and brain development (Figure 1) (Forster et al., 2006). The *RELN* gene is located in an autism susceptibility locus on chromosome 7q22, and triplet GGC repeats in 5′ untranslated regions (5′UTR) in the *RELN* gene have been associated with autism in a Caucasian population (Persico et al., 2001; Skaar et al., 2005) (Table 2). Expression levels of Reelin are decreased in postmortem autism brains (Fatemi et al., 2005). Reelin has also been implicated in pathogenesis of various neuropsychiatric disorders, including schizophrenia, bipolar disorder, lissencephaly, and epilepsy (Fatemi, 2001). Reeler mice, with a 150-kb deletion of the *Reln* gene, exhibit deficits in motor coordination, increased social dominance, and learning and memory impairments (Table 6) (Salinger et al., 2003; Lalonde et al., 2004).

### Transcription factors

Syndromic forms of ASD frequently involve transcription factors. This is likely because defective transcription factors have significant influences on many genes and their downstream molecules, affecting diverse neuronal functions.

MeCP2 (X-linked gene methyl CpG binding protein 2) is one of the best examples. It is a member of a large family of methyl-CpG binding domain (MBD) proteins that selectively binds to methylated DNA and represses gene transcription (Figure 1) (Bienvenu and Chelly, 2006). Its downstream targets encompass ASD-related genes such as *BDNF* and *CDKL5*. Mutations in *MeCP2* are the major cause of Rett syndrome, a progressive neurodevelopmental disorder with autistic features (Amir et al., 1999; Bienvenu and Chelly, 2006; Chahrour and Zoghbi, 2007). *Mecp2*-null mice, an animal model for Rett syndrome, recapitulate most symptomatic traits of Rett syndrome such as respiratory dysfunction, forelimb and hindlimb clasping stereotypy, motor dysfunction, tremor, hypoactivity, anxiety, cognitive impairments, and altered sociability (Table 4) (Shahbazian et al., 2002; Moretti et al., 2005).

*Engrailed-2* is a homeodomain transcription factor associated with ASD. Engrailed-2 is involved in a diverse range of biological processes from embryological development and segmental polarity to brain development and axon guidance (Figure 1) (Brunet et al., 2005; Joyner, 1996). The *Engrailed-2* gene on human chromosome 7q36 is in the autism susceptibility locus, and an association between two intronic SNPs rs1861972 and rs1861973 at *Engrailed-2* locus and ASD has been repeatedly identified in 518 ASD families (Gharani et al., 2004; Benayed et al., 2005) (Table 2). However, these SNPs were not found to be associated with ASD in Han Chinese trios (Wang et al., 2008). This association between *Engrailed-2* and ASD was further confirmed by animal model studies, which showed *Engrailed-2* null mice display social dysfunction and cognitive impairments (Table 6) (Brielmaier et al., 2012). Because Engrailed-2 is expressed upon activation of WNT2-Dvl1 signaling, it appears that the WNT2-Dvl1-Engrailed-2 pathway, which regulates neuronal migration and axonal guidance, may significantly contribute to ASD pathogenesis via neuroanatomical abnormalities. In addition, a base substitution (A218G) mutant of *HOXA1*, another homeobox gene, was reported in autistic individuals (Ingram et al., 2000b), indicating the importance of homeobox genes in normal brain function and ASD.

### Excitatory and inhibitory imbalance

Mutations identified in important synaptic molecules including neuroligins (Jamain et al., 2003), neurexin (Autism Genome Project et al., 2007; Kim et al., 2008) and Shank (Durand et al., 2007; Berkel et al., 2010; Sato et al., 2012) in autistic subjects have prompted investigations into exploring the roles of synaptic dysfunctions in ASD pathogenesis. This “synaptopathy” model of autism has provided much insight into the field (Table 5).

Defects in synaptic proteins would lead to defective transmissions at excitatory and inhibitory synapses, disrupting the E-I balance in postsynaptic neurons, a key mechanism implicated in ASD. In line with this, ASD has been genetically associated with diverse glutamate receptors, including the kainite receptor subunit GluR6 (M867I in the intracytoplasmic C-terminal region of GluR6) (Jamain et al., 2002), the metabotropic glutamate receptor 8 (*GRM8*) (R859C, R1085Q, R1100Q, and intrachromosomal segmental duplication) (Serajee et al., 2003), and the N-methyl-D-aspartic acid receptor (NMDAR) subunit GluN2A (rs1014531) (Barnby et al., 2005), and GluN2B (*de novo* protein truncating and splice mutations) (O'Roak et al., 2012a,b) (Table 2). Decreased levels of glutamine and abnormal levels of glutamate were observed in the plasma of autistic children (Rolf et al., 1993; Moreno-Fuenmayor et al., 1996). In addition, neuropathological studies of postmortem autism brains show perturbations in the glutamate neurotransmitter system (Purcell et al., 2001).

Abnormal GABAergic system is also proposed as a potential mechanism for ASD. Reduced expression levels in a rate-limiting enzyme for GABA synthesis, glutamic acid decarboxylase (GAD), and GABA receptors with altered subunit composition were observed in autistic brains (Fatemi et al., 2002, 2010). Furthermore, linkage disequilibrium and transmission disequilibrium between *GABRB3*, a gene encoding the β3 subunit of GABAα receptors, with Angelman syndrome and autism has been reported (Cook et al., 1998; Bass et al., 2000; Buxbaum et al., 2002) (Table 2).

The serotonergic system would also play a role in ASD pathogenesis by modulating the E-I balance. Serotonin levels in blood or urine are increased in subjects with autism (Cook and Leventhal, 1996; Burgess et al., 2006), and various genes in the serotonin system are linked to autism. Among them are genes encoding the serotonin transporter 5-HTT (transmission disequilibrium at the *5-HTT* locus in 86 autism trios) (Cook et al., 1997), and a rate-limiting enzyme for serotonin synthesis TPH2 (two intronic SNPs rs4341581 and rs11179000 at introns 1 and 4, respectively, have been associated with autism) (Coon et al., 2005) (Table 2).

Neurexins and neuroligins are synaptic cell adhesion molecules enriched at pre- and post-synaptic membranes, respectively (Figure 1) (Craig and Kang, 2007; Sudhof, 2008). Specific interactions between neurexins and neuroligins regulate various aspects of both excitatory and inhibitory synaptic development and function, affecting the E-I balance in postsynaptic neurons. Many mutations in genes encoding neurexins (including hemizygous CNV deletions and missense mutations) and neuroligins (e.g., R451C for *NLGN3* and a frameshift insertion mutation for *NLGN4*) have been associated with ASD, intellectual disability, and schizophrenia (Jamain et al., 2003; Laumonnier et al., 2004; Autism Genome Project et al., 2007; Kim et al., 2008; Walsh et al., 2008) (Table 2). *Neuroligin3* knockin mice with the R451C mutation found in autistic patients recapitulate autistic features including moderately impaired sociability (Table 5) (Tabuchi et al., 2007). Notably, inhibitory transmission was enhanced in the cortical regions of the mutant brains of these mutant mice, suggesting that disrupted E-I balance may contribute to ASD.

*SHANK* family genes encode scaffolding proteins enriched in the postsynaptic density (PSD), a postsynaptic membrane specialization composed of multi-synaptic protein complexes (Figure 1) (Sheng and Kim, 2000). The Shank family contains three known members, Shank1, Shank2 and Shank3, also known as ProSAP3, ProSAP1, and ProSAP2, respectively. The idea that Shanks are involved in the etiology of ASD firstly emerged from Phelan-McDermid syndrome (PMS) or 22q13 deletion syndrome, a neurodevelopmental disorder caused by a microdeletion on chromosome 22 (Boeckers et al., 2002; Wilson et al., 2003; Phelan and McDermid, 2012). The association between *SHANK* and ASD became evident by identifying numerous mutations including *de novo* frameshift, truncating, and missense mutations on *SHANK3* locus in autistic individuals (Durand et al., 2007) (Table 2). Mutations in *SHANK2* and *SHANK1* including *de novo* CNV deletions and missense mutations in Canadian and European populations were also identified in individuals with ASD and intellectual disability (Berkel et al., 2010; Leblond et al., 2012; Sato et al., 2012).

Multiple lines of transgenic mice with *Shank* mutations found in human patients have been reported. *Shank3* heterozygous mice show sociability deficits and reductions in miniature excitatory postsynaptic currents (mEPSC) amplitude and basal synaptic transmission (Bozdagi et al., 2010); mice with deletion of exon 4–9 of *Shank3* are socially impaired and exhibit alterations in dendritic spine morphology and activity-dependent surface expression of AMPARs (Wang et al., 2011); *Shank1*^−/−^ mice display reduced basal synaptic transmission in the hippocampal CA1 region and reduced motor function and anxiety-like behavior, although they show normal sociability (Hung et al., 2008; Silverman et al., 2011); mice expressing Shank2-R462X in hippocampal CA1 neurons exhibit cognitive dysfunction accompanied by reduced mEPSC amplitude and changes in neuronal morphologies (Table 5) (Berkel et al., 2012).

CNTNAP2, a neuronal transmembrane protein, is a member of the neurexin family localized at juxtaparanodes of myelinated axons. Here, CNTNAP2 regulates neuron-glia interactions and potassium channel clustering in myelinated axons (Figure 1) (Poliak et al., 1999). Several SNPs (e.g., rs2710102, rs7794745, rs17236239) and nonsynonymous variants (e.g., I867T) in *CNTNAP2* locus were found to be associated with ASD, language impairment, and cortical dysplasia-focal epilepsy syndrome in humans (Alarcon et al., 2008; Arking et al., 2008; Bakkaloglu et al., 2008; Vernes et al., 2008) (Table 2). In a case-control association study in Spanish autistic patients and controls, however, *CNTNAP2* SNPs rs2710102 and rs7794745 did not associate with ASD (Toma et al., 2013). *Cntnap*2^−/−^ mice recapitulate all three core symptoms of autism, and display abnormal neuronal migration, reduced number of GABAergic interneurons, and abnormal neuronal synchronization (Table 4) (Penagarikano et al., 2011). Excessive grooming and hyperactivity in these mice were restored by the treatment of the antipsychotic risperidone (Table 4), suggesting the possibility of therapeutic intervention for certain symptoms of autism.

SynGAP is a GTPase-activating protein for the Ras small GTPase. SynGAP directly interacts with PSD-95, and negatively regulates the Ras-MAPK signaling pathway, excitatory synapse development, and synaptic transmission and plasticity (Figure 1) (Chen et al., 1998; Kim et al., 1998). In humans, de novo mutations of *SYNGAP1* have been associated with intellectual disability and autism (Hamdan et al., 2011). In addition, a genetic case/control study in European populations associates a rare *de novo* copy number variation in *SYNGAP1* with ASD (Pinto et al., 2010). *Syngap1* heterozygous mice show schizophrenia-like phenotypes including hyperactivity, impaired sensory-motor gating, impaired social memory and fear conditioning, and preference to social isolation (Guo et al., 2009) (Table 5). In a more recent study, *Syngap1* heterozygous mice showed premature dendritic spine development together with enhanced hippocampal excitability and abnormal behaviors, suggesting that over-paced excitatory synaptic development during a critical time window of postnatal brain development causes intellectual disability and ASD (Clement et al., 2012).

Several genes associated with X chromosome-linked intellectual disability (XLID) and synaptic regulations have been associated with ASD. One of them is interleukin 1 receptor accessory protein-like 1 (*IL1RAPL1*) that encodes a synaptic transmembrane protein (Carrie et al., 1999). Recently, a systematic sequencing screen of X chromosomes of ASD-affected individuals has identified a *de novo* frameshift mutation in *IL1RAPL1* (Piton et al., 2008). IL1RAPL1 plays an important role in the formation and stabilization of excitatory synapses by recruiting the scaffolding protein PSD-95 to excitatory postsynaptic sites through the JNK signaling pathway (Figure 1) (Pavlowsky et al., 2010). In addition, IL1RAPL1 induces the presynaptic differentiation through its trans-synaptic interaction with protein tyrosine phosphatase δ (PTPδ) (Figure 1) (Valnegri et al., 2011b; Yoshida et al., 2011). This interaction between IL1RAPL1 and PTPδ recruits RhoGAP2 to the excitatory synapses and induces dendritic spine formation (Valnegri et al., 2011b). Interestingly, IL1RAPL1 regulates the development of inhibitory circuits in the cerebellum, an ASD-related brain region, and disrupts the excitatory and inhibitory balance, as determined by a study using *Il1rapl1*^−/−^ mice (Gambino et al., 2009). These results suggest that IL1RAPL1 is involved in the regulation of excitatory synaptic development and the balance between excitatory and inhibitory synaptic inputs.

Another XLID gene related with ASD is *OLIGOPHRENIN-1* (*OPHN1*), which encodes a GTPase-activating protein that inhibits Rac, Cdc42, and RhoA small GTPases. Since the initial report of the association of a truncation mutation of *OPHN1* with XLID (Billuart et al., 1998a,b), additional studies have associated nonsynonymous rare missense variants in *OPHN1* with ASD (e.g., H705R) and schizophrenia (e.g., M461V) (Piton et al., 2011). OPHN1 regulates dendritic spine morphogenesis through the RhoA signaling pathway (Govek et al., 2004) and activity-dependent synaptic stabilization of AMPA receptors (Nadif Kasri et al., 2009). OPHN1 also interacts with the transcription repressor Rev-erba to regulate expression of circadian oscillators (Valnegri et al., 2011a). Importantly, *Ophn1*^−/−^ mice show immature spine morphology, impaired spatial memory and social behavior, and hyperactivity (Khelfaoui et al., 2007). These results suggest that OPHN1 regulates excitatory synaptic development and function.

*TM4SF2* or *tetraspanin 7* (*TSPAN7*), another X-linked gene which encodes a membrane protein which belongs to transmembrane 4 superfamily (TM4SF), plays important roles in the cell proliferation, activation, growth, adhesion, and migration (Maecker et al., 1997). TM4SF proteins form a complex with integrin, which regulates cell motility and migration by modulating the actin cytoskeleton (Berditchevski and Odintsova, 1999). A balanced translocation and mutations (a nonsense mutation and a P172H missense mutation) of *TM4SF2* was firstly discovered in the individuals with XLID (Zemni et al., 2000). In subsequent studies, the P172H missense mutation was found in individuals with XLID (Maranduba et al., 2004) and ASD (Piton et al., 2011). A microduplication in the locus of *TM4SF2* was revealed, but this duplication was also present in unaffected controls, suggesting that it may be a neutral polymorphism (Cai et al., 2008). In neurons, TM4SF2 regulates excitatory synaptic development and AMPA receptor trafficking by binding to the synaptic PDZ protein PICK1 (Figure 1) (Bassani et al., 2012).

### Synaptic signaling

Disrupted synaptic signaling may be a key determinant of ASD. Components in mGluR- or NMDAR-dependent signaling cascades have recently been implicated in ASD.

Neurofibromin 1 (*NF1*), tuberous sclerosis complex (*TSC1*/*TSC2*), and phosphatase and tensin homolog (*PTEN*) are genes associated with neurological diseases with common autistic symptoms including neurofibromatosis (Rasmussen and Friedman, 2000), tuberous sclerosis (van Slegtenhorst et al., 1997), and Cowden/Lhermitte-Duclos syndrome (Pilarski and Eng, 2004). They are tumor suppressors sharing a common function; they negatively regulate the mammalian target of rapamycin (mTOR) signaling pathway. Although *Tsc1* null mice are embryonically lethal (Wilson et al., 2005), mutant mice with loss of *Tsc1* in cerebellar Purkinje cells display autistic-like behaviors (Tsai et al., 2012), and *Tsc2* heterozygote mice exhibit abnormal social communication (Young et al., 2010); *Nf1* mutant mice show aberrant social transmission of food preference and deficits in hippocampus-dependent learning (Costa et al., 2001, 2002); *Pten* deficient mice show altered social interaction and macrocephaly with hyperactivation of mTOR pathway (Table 4) (Kwon et al., 2006).

Signaling molecules in the downstream of mTOR in the mTOR pathway play crucial roles in ASD pathogenesis. Upon phosphorylation by mTORC1, 4E-BP proteins are detached from eIF4E to promote eIF4E-dependent protein translation (Figure 1) (Richter and Sonenberg, 2005). A SNP at eIF4E promoter region which increases its promotor activity was found in autism patients (Neves-Pereira et al., 2009). Implications of mTOR downstream signaling in ASD were demonstrated as 4E-BP2 knockout mice and eIF4E overexpression mice display autistic-like behaviors. 4E-BP2 knockout mice show enhanced translational control of neuroligins and increased excitatory transmission in the hippocampus (Table 6) (Gkogkas et al., 2013), while eIF4E overexpressing transgenic mice show impaired excitatory/inhibitory balance in the mPFC and increased LTD in the hippocampus and striatum (Table 6) (Santini et al., 2013). Autistic features of these mutant mice were ameliorated by 4EGI-1 infusion, which inhibits the eIF4E–eIF4G interaction.

Fragile X syndrome is the most common cause of intellectual disability and autism. It is mostly caused by the expansion of CGG trinucleotide repeats in the promoter region of the *FMR1* gene, which enhances the methylation of the promoter and represses generation of *FMR1*-encoded protein (FMRP), which binds to target mRNAs and regulates their translation and transport of mRNA into dendrites and synapses (Figure 1) (Bassell and Warren, 2008). In the absence of FMRP, target mRNA translation becomes excessive and uncontrolled, leading to an aberrant activity-dependent protein synthesis. *Fmr1* mutant mice show enhanced protein synthesis-dependent mGluR-mediated LTD and dendritic spine elongation, together with cognitive deficits, social anxiety and impaired social interaction (Table 4) (Bernardet and Crusio, 2006). Interestingly, target molecules of FMRP include Shank3, GluN2A, mTOR, TSC2, NF1, neuroligin2, and neurexin1 (Darnell et al., 2011), which are associated with ASD pathogenesis.

It should be noted that the ASD-related signaling molecules mentioned above are also associated with NMDAR and mGluR signaling pathways. NMDARs and mGluRs play critical roles in the regulation of synaptic function and plasticity at excitatory synapses. NF1 interacts with the NMDAR complex and regulates GluN2A phosphorylation (Figure 1) (Husi et al., 2000). FMRP and TSC have profound effects on mGluR-dependent LTD and protein synthesis, which are upregulated in *Fmr1*^−/y^ mice, while downregulated in *Tsc2*^+/−^ mice (Auerbach et al., 2011). FMRP is also in the downstream of mGluR signaling (Figure 1) (Bassell and Warren, 2008).

Defects in NMDAR function and associated signaling are also observed in nonsyndromic ASD models with *Shank* mutations. Shank proteins are physically connected to both NMDARs and mGluRs, suggesting that Shank may regulate signaling pathways downstream of NMDAR or mGluR activation, and the functional interaction between the two receptors (Figure 1). *Shank2*^−/−^ mice with the deletion of exons 6 and 7 display autistic-like behaviors and reductions in NMDAR function and associated signaling, without affecting mGluR-dependent LTD (Won et al., 2012), while *Shank2*^−/−^ mice with exon 7 deletion show similar behavioral abnormalities with NMDAR hyperfunction (Table 5) (Schmeisser et al., 2012). Although how similar exon deletions in *Shank2* lead to comparable behavioral abnormalities but different changes in NMDAR function remains to be determined, these results point to that Shank2 is an important regulator of NMDAR function, and that NMDAR function and NMDAR-associated signaling are associated with ASD.

### Neuroimmune response

The implication of the immune system on autism was initially proposed in 1976 based on that some autistic children do not have detectable Rubella titers in spite of previous vaccination (Stubbs, 1976). Levels of serum IgG and autoantibodies to neuronal and glial molecules were elevated in autistic patients (Singh et al., 1997; Croonenberghs et al., 2002), proposing involvement of autoimmune responses in autism. In addition, plasma or cerebrospinal fluid (CSF) levels of pro-inflammatory cytokines and chemokines including MCP-1, IL-6, IL-12, IFN-γ and TGFβ1 were increased in autistic individuals (Ashwood and Van de Water, 2004; Ashwood et al., 2006).

Astrocytes and microglia are two glial cell types important for immune responses in the brain as well as regulation of neuronal functions and homeostasis (Fields and Stevens-Graham, 2002). Postmortem analyses demonstrated abnormal glial activation and neuroinflammatory responses in autistic brains (Vargas et al., 2005). Transcriptome analysis of autistic postmortem brain tissues has also revealed upregulation in the expression of genes belonging to immune and inflammatory networks (Voineagu et al., 2011). Reactive astrocytes were also detected in *Cntnap2*^−/−^ brains, a well-established autism model (Penagarikano et al., 2011). These results clearly suggest the association between neuroimmune defects with ASD, although further details remain to be determined.

### Non-genetic models of ASD

Although we have thus far described genetic factors underlying ASD, environmental factors also have strong influences on ASD. Epidemiologic studies suggest that maternal exposure to stress, viral or bacterial infection, thalidomide, and valproic acid can increase the risk for ASD in offspring (Grabrucker, 2012).

Maternal immune activation (MIA) induced by poly(I:C), the synthetic doublestrand RNA polyriboinosinic-polyribocytidilic acid, in pregnant mice leads to the development of core ASD-like phenotypes in the offspring, including impaired sociability, decreased USV, and increased repetitive behaviors (Malkova et al., 2012). MIA by lipopolysaccharide (LPS) treatment during pregnancy can also induces ASD-like phenotypes in rodent offspring, including impaired social interaction (Hava et al., 2006; Kirsten et al., 2010) and reduced USV (Baharnoori et al., 2012). IL-6 is thought to play a critical role in this process, as IL-6 knockout mice do not show poly(I:C) induced social deficits (Smith et al., 2007), and IL-6 levels are significantly elevated in the cerebellum of autistic subjects (Wei et al., 2011). Although further details remain to be determined, the underlying mechanisms may include IL6-dependent regulation of excitatory and inhibitory synaptic transmission and neuroprotection (Sallmann et al., 2000; Biber et al., 2008; Dugan et al., 2009).

Prenatal exposure to teratogens can increase the risk for ASD in animals, as in humans. Thalidomide (THAL) and valproic acid (VPA) cause rat offspring to display brain morphological abnormalities observed in ASD, including altered cerebellar structures and reduced number of cranial motor neurons (Rodier et al., 1997; Ingram et al., 2000a). Behaviorally, VPA-exposed rats show decreases in prepulse inhibition, stereotypy, and social behaviors (Schneider and Przewlocki, 2005). VPA-exposed rats display elevated serotonin levels and abnormal serotonergic neurons (Anderson et al., 1990; Narita et al., 2002; Miyazaki et al., 2005), decreased parvalbumin-positive interneurons in the neocortex (Gogolla et al., 2009), and elevated NMDA receptor levels and enhanced LTP (Rinaldi et al., 2007), suggesting that these mechanisms may contribute to the development of ASD-like phenotypes.

## Potential treatments for ASD

Currently, only two medicines have been approved for ASD by US FDA; risperidone (Risperdal®) and aripiprazole (Abilify®), which act as dopamine/5-HT receptor antagonists (McPheeters et al., 2011). These drugs are useful for correcting irritability and stereotypy, but not sociability defects. Recently, a number of candidate ASD medications for treating social abnormalities have been suggested (Figure 1).

### mGluR positive allosteric modulators

mGluR1 and mGluR5 are group I mGluRs that are postsynaptically expressed in broad brain regions, including the cerebral cortex, striatum, hippocampus, nucleus accumbens, and inferior colliculus (Testa et al., 1995). Upon activation, group I mGluRs enhance calcium release from intracellular stores resulting in neuronal depolarization, augmentation of neuronal excitability, and activation of intracellular signaling cascades such as PKA, PKC, MAPK, ERK, and CREB (Niswender and Conn, 2010). mGluR5 is physically linked to NMDARs via Homer-Shank/ProSAP-GKAP/SAPAP-PSD-95 interactions (Naisbitt et al., 1999; Tu et al., 1999), and is functionally coupled to NMDARs via aforementioned signaling molecules including PKC (Niswender and Conn, 2010). Through these structural and biochemical interactions, mGluR5 activation is thought to potentiate NMDAR function (Awad et al., 2000; Attucci et al., 2001; Mannaioni et al., 2001; Pisani et al., 2001; Alagarsamy et al., 2002; Rosenbrock et al., 2010).

Positive allosteric modulators of mGluR5 receptors were first developed to alleviate symptoms of schizophrenia (Gregory et al., 2011). Although antipsychotics are available for positive symptoms of schizophrenia, such as hallucinations, no medications are currently available for negative symptoms or cognitive impairments. Two main hypotheses have been proposed for schizophrenia: dopaminergic hyperactivity and NMDA hypofunction. Dopaminergic hyperactivity can be treated by dopamine receptor-antagonistic antipsychotics such as risperidone, but NMDA hypofunction is difficult to modulate given the expected side effects of enhancing NMDAR functions.

Therefore, the concept of augmenting NMDAR signaling via mGluR potentiation was proposed to improve negative symptoms of schizophrenia (Uslaner et al., 2009; Stefani and Moghaddam, 2010). mGluR positive allosteric modulators increase the function of NMDAR only when they are occupied by the endogenous ligand glutamate (Figure 1). mGluR positive allosteric modulators have significant advantages over the conventional mGluR agonist, (RS)-3,5-dihydroxyphenylglycine (DHPG). While DHPG has poor specificity toward particular mGluR subtypes, mGluR positive allosteric modulators offer high subtype specificity. Some positive allosteric modulators have high brain blood barrier penetrance, which enables the systemic administration of the drugs. Furthermore, whereas direct mGluR agonists cause rapid receptor desensitization, mGluR positive allosteric modulators potentiate mGluR function with minimal desensitization, because they bind to an allosteric site on the receptor distinct from the orthosteric glutamate binding site. These properties of positive allosteric modulators are predicted to minimize their excitotoxicity and enable high-dose administrations.

A large number of mGluR5 allosteric modulators have been developed (Williams and Lindsley, 2005; Gregory et al., 2011). Of these, CDPPB, ADX47273, MPPA, and VU0092273 readily cross the blood-brain barrier, and CDPPB, particular, has been examined in various behavioral assays and model animals. In CHO (Chinese hamster ovary) cells expressing human mGluR5, CDPPB treatment was shown to enhance mGluR5 activity in a concentration-dependent manner (Kinney et al., 2005). Behaviorally, CDPPB alleviates prepulse inhibition and hyperactivity produced by amphetamine, suggesting that CDPPB could be a potential antipsychotic agent.

Because NMDARs play an essential role in learning and memory, indirect potentiation of NMDARs by mGluR5 positive allosteric modulators may facilitate synaptic plasticity and learning and memory. Indeed, CDPPB and ADX47273 enhance the performance of wild-type mice in the Morris water maze test, a hippocampus-dependent learning and memory paradigm (Ayala et al., 2009). In addition, VU-29 and ADX47273 potentiate two forms of NMDAR-dependent synaptic plasticity—LTP and LTD—in the CA1 region of the hippocampus (Ayala et al., 2009). DFB-treated rats make fewer errors in the Y-maze spatial alternation task (Balschun et al., 2006), and CDPPB and ADX47273 enhance performance in novel object recognition and five-choice serial reaction time tasks (Liu et al., 2008; Uslaner et al., 2009). CDPPB not only improves learning and memory performance of wild-type mice, but also reverses cognitive dysfunction and behavioral inflexibility induced by the NMDAR antagonist MK-801 (Uslaner et al., 2009; Stefani and Moghaddam, 2010). These results suggest that mGluR5 positive allosteric modulators have the potential to improve cognitive impairments associated with brain disorders including schizophrenia and autism.

Indeed, CDPPB has recently shown promise as a potential treatment for ASD. In a study using *Tsc2*^+/−^ mice, a mouse model of tuberous sclerosis characterized by intellectual disability and autism, Mark Bear and colleagues showed that cognitive impairments observed in these mice could be alleviated by CDPPB administration (Table 4) (Auerbach et al., 2011). In addition, social deficits of *Shank2*^−/−^ mice are rescued by CDPPB treatment (Won et al., 2012), implicating hypofunction of mGluRs and NMDARs in social impairment, and suggesting mGluR positive allosteric modulators as novel therapeutics for the treatment of social deficits (Table 5). More recently, CDPPB was shown to reverse defects in social novelty recognition induced by neonatal phencyclidine treatment (Clifton et al., 2012).

### D-cycloserine

Although it is well established that NMDARs critically regulate normal brain functions, the excitotoxicity and poor bioavailability of direct NMDAR agonists have hampered attempts to control brain activity by modulating NMDARs (Quartermain et al., 1994). D-cycloserine is a high-affinity partial agonist of NMDA-coupled, strychnine-insensitive glycine receptors (Figure 1) (Hood et al., 1989). Similar to glycine, D-cycloserine also binds to the glycine site of NMDARs as a partial agonist, potentiating NMDARs by increasing the frequency of channel opening. In addition, because NMDARs are not maximally potentiated by endogenous glycine, there is room for D-cycloserine to further potentiate NMDARs. These properties enable D-cycloserine to act as a positive modulator of NMDARs.

D-cycloserine is a viable drug candidate because it is a partial agonist, displaying efficacy of 40-50% relative to glycine, and has low toxicity and decent bioavailability (Hood et al., 1989). Although the brain penetrance of D-cycloserine is not high, it can nonetheless infiltrate the blood-brain barrier, exerting a peak effect 1 h after intraperitoneal administration (Peterson, 1992). D-cycloserine shows dose-dependent elimination (higher elimination rates with lower doses) and a half-life of 7–15 h in humans and 23 min in mice (lwainsky, 1988; Wlaz et al., 1994).

When glycine levels are low, D-cycloserine amplifies the activity of the NMDAR complex and enhances synaptic plasticity and cognitive function. D-cycloserine alleviates senescence-associated behavioral defects (Flood et al., 1992) and facilitates memory acquisition, consolidation, and retrieval (Quartermain et al., 1994). While low doses (10–20 mg kg^−1^) of D-cycloserine have cognition-enhancing effects (Monahan et al., 1989; Flood et al., 1992; Schuster and Schmidt, 1992; Sirvio et al., 1992; Quartermain et al., 1994), higher doses (>100 mg kg^−1^) exert anticonvulsant effects in tonic convulsion models (Peterson, 1992; Peterson and Schwade, 1993).

Putative effects of D-cycloserine on ASD have been suggested by previous studies. Mice with a *neuroligin1* (*Nlgn1*) deficiency exhibit abnormally increased grooming behavior, and this behavioral anomaly is reversed by D-cycloserine treatment (Table 5) (Blundell et al., 2010). Low-dose D-cycloserine alleviates negative symptoms of schizophrenia-affected individuals (Goff et al., 1999), and reduces social withdrawal and increases social responsiveness in autistic patients (Posey et al., 2004). Moreover, D-cycloserine partially rescues social deficits of *Shank2*^−/−^ mice, supporting the role of NMDAR functionality in autism (Table 5) (Won et al., 2012).

### Benzodiazepines

Recently, benzodiazepines were suggested as putative therapeutic agents for Dravet's syndrome, which is a developmental disorder with myoclonic infantile seizure, ADHD-like inattention and hyperactivity, motor impairment, sleep disorder, anxiety-like behaviors, cognitive defects, autism-like social dysfunction, and restricted interests. Mice heterozygous for a deletion of the α-subunit of the type 1 voltage-gated sodium channel (*Scn1α*^+/−^ mice), an animal model for Dravet's syndrome, recapitulate most features of the disorder, including epilepsy, ataxia, sleep disorder, anxiety-like behaviors, hippocampus-dependent learning impairments, sociability deficits, and excessive repetitive grooming behaviors (Table 4) (Yu et al., 2006; Kalume et al., 2007; Han et al., 2012). In *Scn1α*^+/−^ mouse brains, expression of the voltage-gated sodium channel type-1 (Na_v_1.1) is decreased in GABAergic interneurons, and GABAergic transmission onto postsynaptic neurons was reduced. This would cause a shift in the balance between excitation and inhibition in postsynaptic neurons toward excitation, which may be corrected by stimulating GABA receptors in these neurons. Indeed, it was shown that both behavioral abnormalities and aberrant GABAergic transmission are rescued by low-dose administration of clonazepam (Table 4) (Han et al., 2012).

Clonazepam, a type of benzodiazepine, is a positive allosteric modulator of GABA_A_ receptors that exerts sedative, hypnotic, anxiolytic, anticonvulsant, and muscle relaxing effects (Figure 1) (Rudolph and Knoflach, 2011). Similar to the action of mGluR positive allosteric modulators, clonazepam potentiates GABA signaling only when GABA_A_ receptors are bound by their endogenous ligand, GABA. Therefore, these results indicate that normalization of disrupted E-I balance may be a novel and promising strategy for treating symptoms of ASD.

### mGluR negative allosteric modulators

The potential of mGluR of negative allosteric modulation as a therapeutic strategy in ASD was first proposed based on studies in *Fmr1*^−/y^ mice, an animal model for fragile X syndrome (Bakker et al., 1994). The enhanced mGluR5-dependent LTD and protein synthesis observed in *Fmr1*^−/y^ mice provided a conceptual framework for the mGluR theory of fragile X pathogenesis (Bear et al., 2004; Bassell and Warren, 2008). Synaptic protein synthesis is stimulated by local mRNA translation, a process that depends on group I mGluR activation. FMRP, encoded by the *Fmr1* gene, is a repressor of mRNA translation; thus, mGluR-mediated protein synthesis could be enhanced in the absence of FMRP. Therefore, attempts have been made to correct fragile X syndrome by suppressing abnormally enhanced mGluR5-dependent synaptic plasticity and protein synthesis.

Two approaches have been used to normalize behavioral and neuronal deficits of *Fmr1*^−/y^ mice: genetic crossbreeding with *Tsc2*^+/−^ mice, which exhibit suppressed mGluR activity, and acute administration of an mGluR antagonist (Auerbach et al., 2011). Administering the mGluR5 antagonist 2-methyl-6-(phenylethynyl) pyridine hydrochloride (MPEP) to *Fmr1*^−/y^ mice normalizes defective phenotypes, including cognitive deficits, perturbed mGluR-dependent LTD and protein synthesis, and excessive filopodia-like long and thin spines (Figure 1, Table 4) (Yan et al., 2005; de Vrij et al., 2008). In line with this, mGluR negative allosteric modulators are now in clinical trials for fragile X syndrome patients (Krueger and Bear, 2011).

The therapeutic potential of mGluR antagonists in ASD has also been suggested. Repetitive grooming behaviors in BTBR and valproic acid (VPA) mouse models of autism are significantly alleviated by MPEP treatment (Silverman et al., 2010a; Mehta et al., 2011). Impairments in social interaction of BTBR mice are also ameliorated by MPEP administration (Silverman et al., 2010a). GRN-529, a selective negative allosteric modulator of mGluR5 developed by Pfizer, was shown to fully rescue excessive repetitive grooming behavior and social dysfunction in BTBR mice and jumping stereotypy in C58/J mice (Silverman et al., 2012). These findings suggest that mGluR negative allosteric modulators have novel therapeutic potential in autism, in addition to fragile X syndrome.

### NMDAR antagonists

NMDAR antagonists including amantadine and its close analogue memantine are now in clinical trials for autistic patients (Nightingale, 2012; Spooren et al., 2012). Amantadine and memantine are non-competitive antagonists for NMDARs with multiple clinical uses (Chen et al., 1992; Blanpied et al., 2005). Memantine is currently being used for Alzheimer's disease, while it is also useful for viral infection and Parkinson's disease. Because both drugs are weak NMDAR antagonists with moderate affinity, prolonged receptor blockade during treatment is unlikely to cause significant side effects.

In a double-blind, placebo controlled study, amantadine-treated group show significant improvements in hyperactivity and inappropriate speech (King et al., 2001). Memantine is also effective in improving language and social behavior and clinical global impressions (CGI) scale in autistic patients (Chez et al., 2007; Erickson et al., 2007; Niederhofer, 2007).

With regard to mechanisms of memantine and amantadine underlying the treatment of ASD remains, both medications are highly likely to exert their therapeutic effects by suppressing NMDAR function and modulating excitotoxicity in autistic subjects. However, care should be taken because other possibilities exist. For instance, memantine treatment promotes excitatory synapse formation and maturation and cell adhesion properties of cerebellar granule cells (CGCs) of *Fmr1* knockout mice (Wei et al., 2012). In addition, memantine exerts neuroprotective activities by promoting glia-derived neurotrophic factor (GDNF) release and preventing migroglial inflammatory responses (Wu et al., 2009). Memantine can also act as a non-competitive antagonist for 5-HT receptors (Rammes et al., 2001) and nicotinic acetylcholine receptors (Aracava et al., 2005), while it functions as an agonist for D2 dopamine receptors (Seeman et al., 2008).

### IGF-1

A new approach for alleviating phenotypic traits of ASD in animal models has come from research on Rett syndrome. Rett syndrome is an X-linked neurological disorder caused by mutations in the *MeCP2* gene. MeCP2 is a transcriptional repressor and activator, which binds widely across the genome and influence a large number of genes (Chahrour et al., 2008). One of the best characterized targets of MeCP2 is BDNF, a neurotrophic factor that regulates neuronal development and synaptic plasticity (Figure 1) (Greenberg et al., 2009). *Bdnf* conditional knockout mice show features analogous to Rett syndrome, including smaller brain size and hindlimb-clasping behavior (Chang et al., 2006). Mice with double knockout of *Bdnf* and *MeCP2* show earlier onset of Rett-like symptoms, whereas overexpression of *Bdnf* in *MeCP2* knockout mice delays the onset and relieves the electrophysiological defects of *MeCP2* mutants. Moreover, restoring Bdnf expression through ampakine administration alleviates respiratory problems of *MeCP2* mutant mice (Ogier et al., 2007). Although BDNF appears to have significant effects in Rett syndrome model animals, it poorly penetrates the blood-brain barrier, limiting its therapeutic application.

Another growth factor associated with Rett syndrome is insulin-like growth factor 1 (IGF-1) (Figure 1). IGF-1 is a polypeptide hormone with structural similarity to insulin. While it has a profound effect on overall cell growth, it also plays an important role in regulating neuronal functions by promoting axonal outgrowth (Ozdinler and Macklis, 2006), neuro- and synaptogenesis (O'Kusky et al., 2000), and activity-dependent cortical plasticity (Tropea et al., 2006). IGF-1 binds to IGF-binding proteins (IGFBP1–6), resulting in extension of the half-life of IGF-1 (Hwa et al., 1999). Upon binding to its cognate receptor, IGF-1 activates Ras-MAPK and PI3K-Akt pathways (Fernandez and Torres-Aleman, 2012), signaling cascades that are also activated by BDNF.

Because IGF-1 crosses the blood-brain barrier, it may be a viable alternative to BDNF as a therapeutic agent for Rett syndrome. Indeed, IGF-1 and IGFBP have been implicated in Rett syndrome and autism: IGFBP3 levels are abnormally elevated in MeCP2 mutant mice and Rett syndrome patients (Itoh et al., 2007), and the concentration of IGF-1 in CSF is reduced in autistic individuals (Riikonen et al., 2006). The therapeutic utility of IGF-1 in Rett syndrome was originally suggested by Mriganka Sur and coworkers, who reported that lethality, hypoactivity, and respiratory problems of *MeCP2*-null mice are partially rescued by IGF-1 treatment in association with normalization of impaired spine density, synaptic transmission, and cortical plasticity (Table 4) (Tropea et al., 2009). IGF-1 also reverses the reduction in excitatory synapse number and density of neurons derived from Rett patients (Marchetto et al., 2010).

### Rapamycin

Rapamycin is an immunosuppressant originally identified as an antifungal agent in isolates from *Streptomyces hygroscopicus* (Sehgal et al., 1975; Vezina et al., 1975; Baker et al., 1978; Singh et al., 1979). Rapamycin strongly binds to FK506-binding protein (FKBP); this complex then binds and inhibits mTOR, a serine/threonine kinase implicated in transcription, cytoskeleton dynamics, ubiquitin-dependent protein degradation, autophagy, and membrane trafficking (Figure 1) (Dennis et al., 1999). mTOR signaling has profound effects on neuronal cells in addition to cancer cells (Busaidy et al., 2012), immune cells (Araki et al., 2011), and cells that regulate lifespan (Powers et al., 2006; Harrison et al., 2009). In the nervous system, mTOR regulates axon guidance, dendrite arborization, synaptogenesis, and synaptic plasticity (Troca-Marin et al., 2012).

Perturbations in mTOR signaling have significant impacts on normal brain functions. Patients with Alzheimer's disease and Drosophila tauopathy models show enhanced mTOR signaling in the brain (Li et al., 2005; Khurana et al., 2006). Hyperactivation of the Akt-mTOR pathway is observed in hippocampal neurons of *Ts1Cje* mice, which models Down syndrome (Troca-Marin et al., 2011). Animal models and patients of Parkinson's disease exhibit enhanced levels of REDD1, which inhibits mTOR activity (Malagelada et al., 2006). mTOR is observed in inclusion bodies from Huntington's disease patients and corresponding mouse models (Ravikumar et al., 2004). Importantly, rapamycin treatment alleviates several pathogenic traits observed in *in vivo* and *in vitro* models of Alzheimer's disease (Khurana et al., 2006; Harrison et al., 2009), Parkinson's disease (Pan et al., 2009; Tain et al., 2009), and polyglutamine diseases (Ravikumar et al., 2004; Berger et al., 2006; Pandey et al., 2007).

The therapeutic utility of rapamycin in ASD was suggested in 2008 based on studies in *Tsc2*^+/−^ mice (Ehninger et al., 2008). The mTOR pathway is associated with TSC because TSC1 and TSC2 are upstream inhibitory regulators of mTOR activity (Han and Sahin, 2011). In this study, the learning and memory deficits, lethality, aberrant brain overgrowth, and altered synaptic plasticity of *Tsc2*^+/−^ mice were ameliorated by acute treatment with rapamycin (Table 4). The social dysfunction and behavioral inflexibility of Purkinje cell-specific *Tsc1* mutant mice were also improved by rapamycin (Tsai et al., 2012), further suggesting that rapamycin may be useful in reversing core symptoms of autism.

### Oxytocin

Oxytocin is a nine amino acid neuropeptide hormone synthesized by magnocellular neurons in paraventricular and supraoptic nuclei of the hypothalamus and secreted from the posterior pituitary gland into the circulation (Figure 1). Oxytocin acts through oxytocin receptors (OXTRs), which are abundantly expressed in the amygdala, hippocampus, and hypothalamus (Gould and Zingg, 2003). Oxytocin is associated with various social behaviors including affiliation, maternity, aggression, and pair bonding (Lee et al., 2009; Caldwell, 2012; Feldman, 2012). Given the prominence of oxytocin in the regulation of social behavior, the association of oxytocin with autism pathogenesis has been extensively examined.

Several SNPs of OXTRs are associated with ASD (Wu et al., 2005; Jacob et al., 2007; Yrigollen et al., 2008; Liu et al., 2010). *Oxtr* knockout mice display autistic-like behaviors; they emit fewer USVs upon social isolation, show defects in social recognition and discrimination, and are less aggressive (Table 6) (Takayanagi et al., 2005; Crawley et al., 2007). Supporting the pharmacotherapeutic potential of oxytocin, nasal administration of oxytocin improves social interactions and communications (Andari et al., 2010; Kosaka et al., 2012), reduces repetitive behaviors (Hollander et al., 2003), and enhances social cognition (Hollander et al., 2007) in autism-affected individuals.

## Perspectives

### Homeostatic mechanisms underlying ASD

Tuberous sclerosis and fragile X syndrome are disorders with common symptoms including intellectual disabilities, seizures, and autism. While their genetic determinants are different (*TSC1/TSC2* for tuberous sclerosis and *FMR1* for fragile X syndrome), their gene products both regulate protein synthesis in neurons (Bassell and Warren, 2008; Ehninger et al., 2009). Interestingly, animal models of tuberous sclerosis (*Tsc2*^+/−^ mice) and fragile X syndrome (*Fmr1*^−/y^ mice) display abnormal protein synthesis in opposite directions (Auerbach et al., 2011).

*Tsc2*^+/−^ mice exhibit diminished mGluR-dependent LTD and protein synthesis in the hippocampus, whereas *Fmr1*^−/y^ mice show excessive mGluR-dependent LTD and protein synthesis. Consistent with this, cognitive impairments of the two animal models are corrected by drugs that modulate mGluR5 in the opposite manner (CDPPB for *Tsc2*^+/−^ mice and MPEP for *Fmr1*^−/y^ mice). In addition, crossbreeding of these two mouse lines rescues behavioral impairments and synaptic dysfunctions. These results strongly suggest that mGluR5-mediated synaptic plasticity and protein synthesis in the normal range is important and that deviation in either direction from a normal range can cause brain dysfunctions that yield similar behavioral manifestations.

Another such example comes from two mouse models with different mutations in the same gene. *Shank2*^−/−^ mouse lines lacking exons 6 and 7 (Won et al., 2012) or exon 7 only (Schmeisser et al., 2012), both of which mimic mutations found in humans (Berkel et al., 2010), display similar autistic-like behaviors, but NMDAR function in their brains shows opposite changes: NMDAR hypofunction with exons 6 and 7 deletion and NMDAR hyperfunction with exon 7 deletion. Although further details remain to be explored, this is another example suggesting that NMDAR function in a normal range is important, and that deviations in either directions can lead to similar behavioral abnormalities. Therefore, individuals with mutations in the same gene may have to be carefully diagnosed, for example by high-through sequencing, in order to receive proper treatment.

### Core mechanisms underlying ASD

Given the diverse genetic variations underlying the development of ASD, one obvious challenge in understanding how ASD develops is the wide range of mechanisms associated with it. This diversity poses a serious additional problem in treating ASD: a single medication is likely to cover only a small fraction of individuals with ASD, or a limited spectrum of ASD symptoms.

A related well-known example is the selective effect of risperidone. Risperidone, a dopamine antagonist, is an antipsychotic mainly used to treat schizophrenia and bipolar disorder, and it is currently one of the few FDA-approved medications for autism. The drug mainly ameliorates irritability, hyperactivity, and repetitive and restricted behaviors, but is largely ineffective against social withdrawal and language deficits of autistic individuals (McPheeters et al., 2011). Similarly, risperidone rescues repetitive grooming and hyperactivity, but not social deficits, in *Cntnap2*^−/−^ mice (Penagarikano et al., 2011). Another example is the demonstration that CDPPB rescues only social interaction in *Shank2*-deficient mice but fails to rescue impaired pup retrieval, repetitive jumping, hyperactivity, and anxiety-like behavior (Won et al., 2012). The fact that some medications reverse only selective symptoms/phenotypes of ASD, however, may provide an opportunity to further explore detailed mechanisms underlying particular aspects of ASD etiology. This would, in principle, allow us to dissect and study synaptic or circuit mechanisms that are specifically associated with certain aspects of ASD, such as impaired social interaction, impaired social communication, repetitive behavior, restricted interests, intellectual disability, anxiety, and hyperactivity.

A possible solution to the apparent diversity of ASD-related mechanisms is to identify “core” mechanisms that cover a large fraction of genetic variations, or a broader spectrum of ASD symptoms. The concept of core mechanisms is based on the assumption that a fraction of ASD-related proteins may act together and converge on a common pathway. A possible core mechanism could be excitatory synaptic transmission. Excitatory synaptic development can be regulated by a number of factors including synaptic adhesion molecules, synaptic scaffolding proteins, and actin-regulatory proteins. In addition, excitatory synaptic transmission, which is mainly mediated by AMPAR receptors, can be determined by the regulators of the synaptic trafficking and stabilization of AMPA receptors, and regulated by the signaling pathways in the downstream of NMDA receptors, mGluRs, and monoamine receptors. Another core mechanism could be the E-I balance, which is determined by the relative amounts of excitatory and inhibitory synaptic transmissions, and, together with the excitability of postsynaptic neurons, determines firing patterns of postsynaptic neurons and, subsequently, network activities across the brain. Establishing these core mechanisms, if any, would require rigorous and time-consuming verifications using a range of approaches, including mouse genetics, electrophysiology, and behavior.

### Integrating three aspects of ASD research: human genetics, mouse models, and potential treatments

An important starting point for ASD research using mouse models would be to select best possible genetic variations that can provide us decent insights into the underlying mechanisms and potential treatments. Luckily, a large number of ASD-related papers are being published each year (i.e., ~2500 papers in 2012 when “autism” was used as a search key word in PubMed). These publications, which use diverse genetic and genomic approaches and often large size samples, have identified overlapping genes and mutations, which are likely to have greater influences on the development of ASD. Characterization of transgenic mouse lines that carry these frequent genetic variations would help us efficiently obtain ASD mechanisms with a greater impact.

The synaptic and circuit mechanisms derived from ASD mouse model researches would provide clues to the ways to rescue synaptic/circuit phenotypes and ASD-like behaviors in mice. Given that there is no FDA-approved treatment for social deficits in ASD as of now, these rescue results will only be useful in supporting that the candidate mechanisms are indeed causing the ASD-like phenotypes in mice. Importantly, however, some of these mechanism-based rescues may serve as the basis for clinical trials. Eventually, some of the clinically verified medications may return to basic ASD research and be used to identify additional ASD mouse models with similar or novel underlying mechanisms, which will help us understand a bigger picture, where many synaptic and circuit mechanisms act together and converge into more comprehensive mechanisms.

### Conflict of interest statement

The authors declare that the research was conducted in the absence of any commercial or financial relationships that could be construed as a potential conflict of interest.

## References

[B1] Abrahams B. S. Geschwind D. H. (2008). Advances in autism genetics: on the threshold of a new neurobiology. Nat. Rev. Genet. 9, 341–355 10.1038/nrg2346 18414403 PMC2756414

[B2] Alagarsamy S. Rouse S. T. Junge C. Hubert G. W. Gutman D. Smith Y. (2002). NMDA-induced phosphorylation and regulation of mGluR5. Pharmacol. Biochem. Behav. 73, 299–306 10.1016/S0091-3057(02)00826-2 12117583

[B3] Alarcon M. Abrahams B. S. Stone J. L. Duvall J. A. Perederiy J. V. Bomar J. M. (2008). Linkage, association, and gene-expression analyses identify CNTNAP2 as an autism-susceptibility gene. Am. J. Hum. Genet. 82, 150–159 10.1016/j.ajhg.2007.09.005 18179893 PMC2253955

[B4] Allen G. Courchesne E. (2003). Differential effects of developmental cerebellar abnormality on cognitive and motor functions in the cerebellum: an fMRI study of autism. Am. J. Psychiatry 160, 262–273 10.1176/appi.ajp.160.2.262 12562572

[B5] Allen G. Muller R. A. Courchesne E. (2004). Cerebellar function in autism: functional magnetic resonance image activation during a simple motor task. Biol. Psychiatry 56, 269–278 10.1016/j.biopsych.2004.06.005 15312815

[B6] Amir R. E. Van den Veyver I. B. Wan M. Tran C. Q. Francke U. Zoghbi H. Y. (1999). Rett syndrome is caused by mutations in X-linked MECP2, encoding methyl-CpG-binding protein 2. Nat. Genet. 23, 185–188 10.1038/13810 10508514

[B7] Andari E. Duhamel J. R. Zalla T. Herbrecht E. Leboyer M. Sirigu A. (2010). Promoting social behavior with oxytocin in high-functioning autism spectrum disorders. Proc. Natl. Acad. Sci. U.S.A. 107, 4389–4394 10.1073/pnas.0910249107 20160081 PMC2840168

[B8] Anderson G. M. Horne W. C. Chatterjee D. Cohen D. J. (1990). The hyperserotonemia of autism. Ann. N.Y. Acad. Sci. 600, 331–340 discussion: 341–332. 10.1111/j.1749-6632.1990.tb16893.x 2252319

[B9] Anney R. Klei L. Pinto D. Regan R. Conroy J. Magalhaes T. R. (2010). A genome-wide scan for common alleles affecting risk for autism. Hum. Mol. Genet. 19, 4072–4082 10.1093/hmg/ddq307 20663923 PMC2947401

[B10] Aracava Y. Pereira E. F. Maelicke A. Albuquerque E. X. (2005). Memantine blocks alpha7^*^ nicotinic acetylcholine receptors more potently than n-methyl-D-aspartate receptors in rat hippocampal neurons. J. Pharmacol. Exp. Ther. 312, 1195–1205 10.1124/jpet.104.077172 15522999

[B11] Araki K. Ellebedy A. H. Ahmed R. (2011). TOR in the immune system. Curr. Opin. Cell Biol. 23, 707–715 10.1016/j.ceb.2011.08.006 21925855 PMC3241972

[B12] Arking D. E. Cutler D. J. Brune C. W. Teslovich T. M. West K. Ikeda M. (2008). A common genetic variant in the neurexin superfamily member CNTNAP2 increases familial risk of autism. Am. J. Hum. Genet. 82, 160–164 10.1016/j.ajhg.2007.09.015 18179894 PMC2253968

[B13] Ashwood P. Van de Water J. (2004). A review of autism and the immune response. Clin. Dev. Immunol. 11, 165–174 10.1080/10446670410001722096 15330453 PMC2270714

[B14] Ashwood P. Wills S. Van de Water J. (2006). The immune response in autism: a new frontier for autism research. J. Leukoc. Biol. 80, 1–15 10.1189/jlb.1205707 16698940

[B15] Attucci S. Carla V. Mannaioni G. Moroni F. (2001). Activation of type 5 metabotropic glutamate receptors enhances NMDA responses in mice cortical wedges. Br. J. Pharmacol. 132, 799–806 10.1038/sj.bjp.0703904 11181420 PMC1572635

[B16] Auerbach B. D. Osterweil E. K. Bear M. F. (2011). Mutations causing syndromic autism define an axis of synaptic pathophysiology. Nature 480, 63–68 10.1038/nature10658 22113615 PMC3228874

[B17] Autism Developmental Disabilities Monitoring Network Surveillance Year 2008 Principal Investigators; Centers for Disease Control and Prevention. (2007). Prevalence of autism spectrum disorders–autism and developmental disabilities monitoring network, 14 sites, United States. MMWR Surveill. Summ. 56, 12–28 17287715

[B18] Autism Developmental Disabilities Monitoring Network Surveillance Year 2008 Principal Investigators; Centers for Disease Control and Prevention. (2009). Prevalence of autism spectrum disorders - Autism and developmental disabilities monitoring network, United States, 2006. MMWR Surveill. Summ. 58, 1–20 20023608

[B19] Autism Genome Project, C. Szatmari P. Paterson A. D. Zwaigenbaum L. Roberts W. Brian J. (2007). Mapping autism risk loci using genetic linkage and chromosomal rearrangements. Nat. Genet. 39, 319–328 10.1038/ng1985 17322880 PMC4867008

[B20] Awad H. Hubert G. W. Smith Y. Levey A. I. Conn P. J. (2000). Activation of metabotropic glutamate receptor 5 has direct excitatory effects and potentiates NMDA receptor currents in neurons of the subthalamic nucleus. J. Neurosci. 20, 7871–7879 11050106 10.1523/JNEUROSCI.20-21-07871.2000 PMC6772731

[B21] Ayala J. E. Chen Y. Banko J. L. Sheffler D. J. Williams R. Telk A. N. (2009). mGluR5 positive allosteric modulators facilitate both hippocampal LTP and LTD and enhance spatial learning. Neuropsychopharmacology 34, 2057–2071 10.1038/npp.2009.30 19295507 PMC2884290

[B22] Aylward E. H. Minshew N. J. Goldstein G. Honeycutt N. A. Augustine A. M. Yates K. O. (1999). MRI volumes of amygdala and hippocampus in non-mentally retarded autistic adolescents and adults. Neurology 53, 2145–2150 10.1212/WNL.53.9.2145 10599796

[B23] Bah J. Quach H. Ebstein R. P. Segman R. H. Melke J. Jamain S. (2004). Maternal transmission disequilibrium of the glutamate receptor GRIK2 in schizophrenia. Neuroreport 15, 1987–1991 10.1097/00001756-200408260-00031 15305151

[B24] Baharnoori M. Bhardwaj S. K. Srivastava L. K. (2012). Neonatal behavioral changes in rats with gestational exposure to lipopolysaccharide: a prenatal infection model for developmental neuropsychiatric disorders. Schizophr. Bull. 38, 444–456 10.1093/schbul/sbq098 20805287 PMC3329978

[B25] Baker H. Sidorowicz A. Sehgal S. N. Vezina C. (1978). Rapamycin (AY-22,989), a new antifungal antibiotic. III. *In vitro* and *in vivo* evaluation. J. Antibiot. 31, 539–545 10.7164/antibiotics.31.539 28309

[B26] Bakkaloglu B. O'Roak B. J. Louvi A. Gupta A. R. Abelson J. F. Morgan T. M. (2008). Molecular cytogenetic analysis and resequencing of contactin associated protein-like 2 in autism spectrum disorders. Am. J. Hum. Genet. 82, 165–173 10.1016/j.ajhg.2007.09.017 18179895 PMC2253974

[B27] Bakker C. E. Verheij C. Willemsen R. Helm R.v.d. Oerlemans F. Vermey M. (1994). Fmr1 knockout mice: a model to study fragile X mental retardation. The Dutch-Belgian Fragile X Consortium. Cell 78, 23–33 8033209

[B28] Balschun D. Zuschratter W. Wetzel W. (2006). Allosteric enhancement of metabotropic glutamate receptor 5 function promotes spatial memory. Neuroscience 142, 691–702 10.1016/j.neuroscience.2006.06.043 16890368

[B29] Barnby G. Abbott A. Sykes N. Morris A. Weeks D. E. Mott R. (2005). Candidate-gene screening and association analysis at the autism-susceptibility locus on chromosome 16p: evidence of association at GRIN2A and ABAT. Am. J. Hum. Genet. 76, 950–966 10.1086/430454 15830322 PMC1196454

[B30] Barnea-Goraly N. Kwon H. Menon V. Eliez S. Lotspeich L. Reiss A. L. (2004). White matter structure in autism: preliminary evidence from diffusion tensor imaging. Biol. Psychiatry 55, 323–326 10.1016/j.biopsych.2003.10.022 14744477

[B31] Baron-Cohen S. Belmonte M. K. (2005). Autism: a window onto the development of the social and the analytic brain. Annu. Rev. Neurosci. 28, 109–126 10.1146/annurev.neuro.27.070203.144137 16033325

[B32] Bass M. P. Menold M. M. Wolpert C. M. Donnelly S. L. Ravan S. A. Hauser E. R. (2000). Genetic studies in autistic disorder and chromosome 15. Neurogenetics 2, 219–226 10.1007/s100489900081 10983717

[B33] Bassani S. Cingolani L. A. Valnegri P. Folci A. Zapata J. Gianfelice A. (2012). The X-linked intellectual disability protein TSPAN7 regulates excitatory synapse development and AMPAR trafficking. Neuron 73, 1143–1158 10.1016/j.neuron.2012.01.021 22445342 PMC3314997

[B34] Bassell G. J. Warren S. T. (2008). Fragile X syndrome: loss of local mRNA regulation alters synaptic development and function. Neuron 60, 201–214 10.1016/j.neuron.2008.10.004 18957214 PMC3691995

[B35] Baudouin S. J. Gaudias J. Gerharz S. Hatstatt L. Zhou K. Punnakkal P. (2012). Shared synaptic pathophysiology in syndromic and nonsyndromic rodent models of autism. Science 338, 128–132 10.1126/science.1224159 22983708

[B36] Bear M. F. Huber K. M. Warren S. T. (2004). The mGluR theory of fragile X mental retardation. Trends Neurosci. 27, 370–377 10.1016/j.tins.2004.04.009 15219735

[B37] Benayed R. Gharani N. Rossman I. Mancuso V. Lazar G. Kamdar S. (2005). Support for the homeobox transcription factor gene ENGRAILED 2 as an autism spectrum disorder susceptibility locus. Am. J. Hum. Genet. 77, 851–868 10.1086/497705 16252243 PMC1271392

[B38] Berditchevski F. Odintsova E. (1999). Characterization of integrin-tetraspanin adhesion complexes: role of tetraspanins in integrin signaling. J. Cell Biol. 146, 477–492 10.1083/jcb.146.2.477 10427099 PMC2156181

[B39] Berger Z. Ravikumar B. Menzies F. M. Oroz L. G. Underwood B. R. Pangalos M. N. (2006). Rapamycin alleviates toxicity of different aggregate-prone proteins. Hum. Mol. Genet. 15, 433–442 10.1093/hmg/ddi458 16368705

[B40] Berkel S. Marshall C. R. Weiss B. Howe J. Roeth R. Moog U. (2010). Mutations in the SHANK2 synaptic scaffolding gene in autism spectrum disorder and mental retardation. Nat. Genet. 42, 489–491 10.1038/ng.589 20473310

[B41] Berkel S. Tang W. Trevino M. Vogt M. Obenhaus H. A. Gass P. (2012). Inherited and de novo SHANK2 variants associated with autism spectrum disorder impair neuronal morphogenesis and physiology. Hum. Mol. Genet. 21, 344–357 10.1093/hmg/ddr470 21994763 PMC3276277

[B42] Bernardet M. Crusio W. E. (2006). Fmr1 KO mice as a possible model of autistic features. ScientificWorldJournal 6, 1164–1176 10.1100/tsw.2006.220 16998604 PMC5917219

[B43] Biber K. Pinto-Duarte A. Wittendorp M. C. Dolga A. M. Fernandes C. C. Von Frijtag Drabbe Kunzel J. (2008). Interleukin-6 upregulates neuronal adenosine A1 receptors: implications for neuromodulation and neuroprotection. Neuropsychopharmacology 33, 2237–2250 17987062 10.1038/sj.npp.1301612

[B44] Bienvenu T. Chelly J. (2006). Molecular genetics of Rett syndrome: when DNA methylation goes unrecognized. Nat. Rev. Genet. 7, 415–426 10.1038/nrg1878 16708070

[B45] Billuart P. Bienvenu T. Ronce N. des Portes V. Vinet M. C. Zemni R. (1998a). Oligophrenin 1 encodes a rho-GAP protein involved in X-linked mental retardation. Pathol. Biol. 46, 678 9885813

[B46] Billuart P. Bienvenu T. Ronce N. des Portes V. Vinet M. C. Zemni R. (1998b). Oligophrenin-1 encodes a rhoGAP protein involved in X-linked mental retardation. Nature 392, 923–926 9582072 10.1038/31940

[B47] Blanpied T. A. Clarke R. J. Johnson J. W. (2005). Amantadine inhibits NMDA receptors by accelerating channel closure during channel block. J. Neurosci. 25, 3312–3322 10.1523/JNEUROSCI.4262-04.2005 15800186 PMC6724906

[B48] Blundell J. Blaiss C. A. Etherton M. R. Espinosa F. Tabuchi K. Walz C. (2010). Neuroligin-1 deletion results in impaired spatial memory and increased repetitive behavior. J. Neurosci. 30, 2115–2129 10.1523/JNEUROSCI.4517-09.2010 20147539 PMC2824441

[B49] Boeckers T. M. Bockmann J. Kreutz M. R. Gundelfinger E. D. (2002). ProSAP/Shank proteins - a family of higher order organizing molecules of the postsynaptic density with an emerging role in human neurological disease. J. Neurochem. 81, 903–910 10.1046/j.1471-4159.2002.00931.x 12065602

[B50] Bolton P. F. Roobol M. Allsopp L. Pickles A. (2001). Association between idiopathic infantile macrocephaly and autism spectrum disorders. Lancet 358, 726–727 10.1016/S0140-6736(01)05903-7 11551582

[B51] Bozdagi O. Sakurai T. Papapetrou D. Wang X. Dickstein D. L. Takahashi N. (2010). Haploinsufficiency of the autism-associated Shank3 gene leads to deficits in synaptic function, social interaction, and social communication. Mol. Autism 1, 15 10.1186/2040-2392-1-15 21167025 PMC3019144

[B52] Brennand K. J. Simone A. Jou J. Gelboin-Burkhart C. Tran N. Sangar S. (2011). Modelling schizophrenia using human induced pluripotent stem cells. Nature 473, 221–225 10.1038/nature09915 21490598 PMC3392969

[B53] Brielmaier J. Matteson P. G. Silverman J. L. Senerth J. M. Kelly S. Genestine M. (2012). Autism-relevant social abnormalities and cognitive deficits in engrailed-2 knockout mice. PLoS ONE 7:e40914 10.1371/journal.pone.0040914 22829897 PMC3400671

[B54] Brunet I. Weinl C. Piper M. Trembleau A. Volovitch M. Harris W. (2005). The transcription factor Engrailed-2 guides retinal axons. Nature 438, 94–98 10.1038/nature04110 16267555 PMC3785142

[B55] Burgess N. K. Sweeten T. L. McMahon W. M. Fujinami R. S. (2006). Hyperserotoninemia and altered immunity in autism. J. Autism Dev. Disord. 36, 697–704 10.1007/s10803-006-0100-7 16614791

[B56] Busaidy N. L. Farooki A. Dowlati A. Perentesis J. P. Dancey J. E. Doyle L. A. (2012). Management of metabolic effects associated with anticancer agents targeting the PI3K-Akt-mTOR pathway. J. Clin. Oncol. 30, 2919–2928 10.1200/JCO.2011.39.7356 22778315 PMC3410405

[B57] Buxbaum J. D. Silverman J. M. Smith C. J. Greenberg D. A. Kilifarski M. Reichert J. (2002). Association between a GABRB3 polymorphism and autism. Mol. Psychiatry 7, 311–316 10.1038/sj.mp.4001011 11920158

[B58] Cai G. Edelmann L. Goldsmith J. E. Cohen N. Nakamine A. Reichert J. G. (2008). Multiplex ligation-dependent probe amplification for genetic screening in autism spectrum disorders: efficient identification of known microduplications and identification of a novel microduplication in ASMT. BMC Med. Genomics 1:50 10.1186/1755-8794-1-50 18925931 PMC2588447

[B59] Caldwell H. K. (2012). Neurobiology of sociability. Adv. Exp. Med. Biol. 739, 187–205 10.1007/978-1-4614-1704-0_12 22399403 PMC4146394

[B60] Campbell D. B. D'Oronzio R. Garbett K. Ebert P. J. Mirnics K. Levitt P. (2007). Disruption of cerebral cortex MET signaling in autism spectrum disorder. Ann. Neurol. 62, 243–250 10.1002/ana.21180 17696172

[B61] Campbell D. B. Sutcliffe J. S. Ebert P. J. Militerni R. Bravaccio C. Trillo S. (2006). A genetic variant that disrupts MET transcription is associated with autism. Proc. Natl. Acad. Sci. U.S.A. 103, 16834–16839 10.1073/pnas.0605296103 17053076 PMC1838551

[B62] Carrie A. Jun L. Bienvenu T. Vinet M. C. McDonell N. Couvert P. (1999). A new member of the IL-1 receptor family highly expressed in hippocampus and involved in X-linked mental retardation. Nat. Genet. 23, 25–31 10.1038/12623 10471494

[B63] Caspi A. Sugden K. Moffitt T. E. Taylor A. Craig I. W. Harrington H. (2003). Influence of life stress on depression: moderation by a polymorphism in the 5-HTT gene. Science 301, 386–389 10.1126/science.1083968 12869766

[B64] Chahrour M. Jung S. Y. Shaw C. Zhou X. Wong S. T. Qin J. (2008). MeCP2, a key contributor to neurological disease, activates and represses transcription. Science 320, 1224–1229 10.1126/science.1153252 18511691 PMC2443785

[B65] Chahrour M. Zoghbi H. Y. (2007). The story of Rett syndrome: from clinic to neurobiology. Neuron 56, 422–437 10.1016/j.neuron.2007.10.001 17988628

[B66] Chang Q. Khare G. Dani V. Nelson S. Jaenisch R. (2006). The disease progression of Mecp2 mutant mice is affected by the level of BDNF expression. Neuron 49, 341–348 10.1016/j.neuron.2005.12.027 16446138

[B67] Chen H. J. Rojas-Soto M. Oguni A. Kennedy M. B. (1998). A synaptic Ras-GTPase activating protein (p135 SynGAP) inhibited by CaM kinase II. Neuron 20, 895–904 10.1016/S0896-6273(00)80471-7 9620694

[B68] Chen H. S. Pellegrini J. W. Aggarwal S. K. Lei S. Z. Warach S. Jensen F. E. (1992). Open-channel block of N-methyl-D-aspartate (NMDA) responses by memantine: therapeutic advantage against NMDA receptor-mediated neurotoxicity. J. Neurosci. 12, 4427–4436 1432103 10.1523/JNEUROSCI.12-11-04427.1992 PMC6576016

[B69] Chez M. G. Burton Q. Dowling T. Chang M. Khanna P. Kramer C. (2007). Memantine as adjunctive therapy in children diagnosed with autistic spectrum disorders: an observation of initial clinical response and maintenance tolerability. J. Child Neurol. 22, 574–579 10.1177/0883073807302611 17690064

[B70] Chien Y. L. Wu Y. Y. Chiu Y. N. Liu S. K. Tsai W. C. Lin P. I. (2011). Association study of the CNS patterning genes and autism in Han Chinese in Taiwan. Prog. Neuropsychopharmacol. Biol. Psychiatry 35, 1512–1517 10.1016/j.pnpbp.2011.04.010 21575668

[B71] Cisternas F. A. Vincent J. B. Scherer S. W. Ray P. N. (2003). Cloning and characterization of human CADPS and CADPS2, new members of the Ca2+-dependent activator for secretion protein family. Genomics 81, 279–291 10.1016/S0888-7543(02)00040-X 12659812

[B72] Clement J. P. Aceti M. Creson T. K. Ozkan E. D. Shi Y. Reish N. J. (2012). Pathogenic SYNGAP1 mutations impair cognitive development by disrupting maturation of dendritic spine synapses. Cell 151, 709–723 10.1016/j.cell.2012.08.045 23141534 PMC3500766

[B73] Clifton N. E. Morisot N. Girardon S. Millan M. J. Loiseau F. (2012). Enhancement of social novelty discrimination by positive allosteric modulators at metabotropic glutamate 5 receptors: adolescent administration prevents adult-onset deficits induced by neonatal treatment with phencyclidine. Psychopharmacology 225, 579–594 10.1007/s00213-012-2845-3 22983144

[B74] Connolly A. M. Chez M. Streif E. M. Keeling R. M. Golumbek P. T. Kwon J. M. (2006). Brain-derived neurotrophic factor and autoantibodies to neural antigens in sera of children with autistic spectrum disorders, Landau-Kleffner syndrome, and epilepsy. Biol. Psychiatry 59, 354–363 10.1016/j.biopsych.2005.07.004 16181614

[B75] Cook E. H. Jr. Courchesne R. Lord C. Cox N. J. Yan S. Lincoln A. (1997). Evidence of linkage between the serotonin transporter and autistic disorder. Mol. Psychiatry 2, 247–250 9152989 10.1038/sj.mp.4000266

[B76] Cook E. H. Jr. Courchesne R. Y. Cox N. J. Lord C. Gonen D. Guter S. J. (1998). Linkage-disequilibrium mapping of autistic disorder, with 15q11-13 markers. Am. J. Hum. Genet. 62, 1077–1083 9545402 10.1086/301832 PMC1377089

[B77] Cook E. H. Leventhal B. L. (1996). The serotonin system in autism. Curr. Opin. Pediatr. 8, 348–354 10.1097/00008480-199608000-00008 9053096

[B78] Coon H. Dunn D. Lainhart J. Miller J. Hamil C. Battaglia A. (2005). Possible association between autism and variants in the brain-expressed tryptophan hydroxylase gene (TPH2). Am. J. Med. Genet. B Neuropsychiatr. Genet. 135B, 42–46 15768392 10.1002/ajmg.b.30168

[B79] Costa R. M. Federov N. B. Kogan J. H. Murphy G. G. Stern J. Ohno M. (2002). Mechanism for the learning deficits in a mouse model of neurofibromatosis type 1. Nature 415, 526–530 10.1038/nature711 11793011

[B80] Costa R. M. Yang T. Huynh D. P. Pulst S. M. Viskochil D. H. Silva A. J. (2001). Learning deficits, but normal development and tumor predisposition, in mice lacking exon 23a of Nf1. Nat. Genet. 27, 399–405 10.1038/86898 11279521

[B81] Courchesne E. (1997). Brainstem, cerebellar and limbic neuroanatomical abnormalities in autism. Curr. Opin. Neurobiol. 7, 269–278 10.1016/S0959-4388(97)80016-5 9142760

[B82] Courchesne E. (2002). Abnormal early brain development in autism. Mol. Psychiatry 7(Suppl. 2), S21–S23 12142938 10.1038/sj.mp.4001169

[B83] Courchesne E. Carper R. Akshoomoff N. (2003). Evidence of brain overgrowth in the first year of life in autism. JAMA 290, 337–344 10.1001/jama.290.3.337 12865374

[B84] Courchesne E. Pierce K. Schumann C. M. Redcay E. Buckwalter J. A. Kennedy D. P. (2007). Mapping early brain development in autism. Neuron 56, 399–413 10.1016/j.neuron.2007.10.016 17964254

[B85] Craig A. M. Kang Y. (2007). Neurexin-neuroligin signaling in synapse development. Curr. Opin. Neurobiol. 17, 43–52 10.1016/j.conb.2007.01.011 17275284 PMC2820508

[B86] Crawley J. N. Chen T. Puri A. Washburn R. Sullivan T. L. Hill J. M. (2007). Social approach behaviors in oxytocin knockout mice: comparison of two independent lines tested in different laboratory environments. Neuropeptides 41, 145–163 10.1016/j.npep.2007.02.002 17420046

[B87] Croonenberghs J. Wauters A. Devreese K. Verkerk R. Scharpe S. Bosmans E. (2002). Increased serum albumin, gamma globulin, immunoglobulin IgG, and IgG2 and IgG4 in autism. Psychol. Med. 32, 1457–1463 10.1017/S0033291702006037 12455944

[B88] Darnell J. C. Van Driesche S. J. Zhang C. Hung K. Y. Mele A. Fraser C. E. (2011). FMRP stalls ribosomal translocation on mRNAs linked to synaptic function and autism. Cell 146, 247–261 10.1016/j.cell.2011.06.013 21784246 PMC3232425

[B89] de Vrij F. M. Levenga J. van der Linde H. C. Koekkoek S. K. De Zeeuw C. I. Nelson D. L. (2008). Rescue of behavioral phenotype and neuronal protrusion morphology in Fmr1 KO mice. Neurobiol. Dis. 31, 127–132 10.1016/j.nbd.2008.04.002 18571098 PMC2481236

[B90] Demontis D. Nyegaard M. Buttenschon H. N. Hedemand A. Pedersen C. B. Grove J. (2011). Association of GRIN1 and GRIN2A-D with schizophrenia and genetic interaction with maternal herpes simplex virus-2 infection affecting disease risk. Am. J. Med. Genet. B Neuropsychiatr. Genet. 156B, 913–922 21919190 10.1002/ajmg.b.31234

[B91] Dennis P. B. Fumagalli S. Thomas G. (1999). Target of rapamycin (TOR): balancing the opposing forces of protein synthesis and degradation. Curr. Opin. Genet. Dev. 9, 49–54 10.1016/S0959-437X(99)80007-0 10072357

[B92] Devlin B. Scherer S. W. (2012). Genetic architecture in autism spectrum disorder. Curr. Opin. Genet. Dev. 22, 229–237 10.1016/j.gde.2012.03.002 22463983

[B93] Dugan L. L. Ali S. S. Shekhtman G. Roberts A. J. Lucero J. Quick K. L. (2009). IL-6 mediated degeneration of forebrain GABAergic interneurons and cognitive impairment in aged mice through activation of neuronal NADPH oxidase. PLoS ONE 4:e5518 10.1371/journal.pone.0005518 19436757 PMC2678193

[B94] Durand C. M. Betancur C. Boeckers T. M. Bockmann J. Chaste P. Fauchereau F. (2007). Mutations in the gene encoding the synaptic scaffolding protein SHANK3 are associated with autism spectrum disorders. Nat. Genet. 39, 25–27 10.1038/ng1933 17173049 PMC2082049

[B95] Ehninger D. de Vries P. J. Silva A. J. (2009). From mTOR to cognition: molecular and cellular mechanisms of cognitive impairments in tuberous sclerosis. JIDR 53, 838–851 10.1111/j.1365-2788.2009.01208.x 19694899 PMC2844770

[B96] Ehninger D. Han S. Shilyansky C. Zhou Y. Li W. Kwiatkowski D. J. (2008). Reversal of learning deficits in a Tsc2+/- mouse model of tuberous sclerosis. Nat. Med. 14, 843–848 10.1038/nm1788 18568033 PMC2664098

[B97] Erickson C. A. Posey D. J. Stigler K. A. Mullett J. Katschke A. R. McDougle C. J. (2007). A retrospective study of memantine in children and adolescents with pervasive developmental disorders. Psychopharmacology 191, 141–147 10.1007/s00213-006-0518-9 17016714

[B98] Fatemi S. H. (2001). Reelin mutations in mouse and man: from reeler mouse to schizophrenia, mood disorders, autism and lissencephaly. Mol. Psychiatry 6, 129–133 10.1038/sj.mp.4000129 11317213

[B99] Fatemi S. H. Halt A. R. Stary J. M. Kanodia R. Schulz S. C. Realmuto G. R. (2002). Glutamic acid decarboxylase 65 and 67 kDa proteins are reduced in autistic parietal and cerebellar cortices. Biol. Psychiatry 52, 805–810 10.1016/S0006-3223(02)01430-0 12372652

[B100] Fatemi S. H. Reutiman T. J. Folsom T. D. Rooney R. J. Patel D. H. Thuras P. D. (2010). mRNA and protein levels for GABAAalpha4, alpha5, beta1 and GABABR1 receptors are altered in brains from subjects with autism. J. Autism Dev. Disord. 40, 743–750 10.1007/s10803-009-0924-z 20066485 PMC2865581

[B101] Fatemi S. H. Snow A. V. Stary J. M. Araghi-Niknam M. Reutiman T. J. Lee S. (2005). Reelin signaling is impaired in autism. Biol. Psychiatry 57, 777–787 10.1016/j.biopsych.2004.12.018 15820235

[B102] Feldman R. (2012). Oxytocin and social affiliation in humans. Horm. Behav. 61, 380–391 10.1016/j.yhbeh.2012.01.008 22285934

[B103] Fernandez A. M. Torres-Aleman I. (2012). The many faces of insulin-like peptide signalling in the brain. Nat. Rev. Neurosci. 13, 225–239 10.1038/nrn3209 22430016

[B104] Feucht M. Fuchs K. Pichlbauer E. Hornik K. Scharfetter J. Goessler R. (1999). Possible association between childhood absence epilepsy and the gene encoding GABRB3. Biol. Psychiatry 46, 997–1002 10.1016/S0006-3223(99)00039-6 10509183

[B105] Fields R. D. Stevens-Graham B. (2002). New insights into neuron-glia communication. Science 298, 556–562 10.1126/science.298.5593.556 12386325 PMC1226318

[B106] Flood J. F. Morley J. E. Lanthorn T. H. (1992). Effect on memory processing by D-cycloserine, an agonist of the NMDA/glycine receptor. Eur. J. Pharmacol. 221, 249–254 10.1016/0014-2999(92)90709-D 1330624

[B107] Folstein S. E. Rosen-Sheidley B. (2001). Genetics of autism: complex aetiology for a heterogeneous disorder. Nat. Rev. Genet. 2, 943–955 10.1038/35103559 11733747

[B108] Fombonne E. Roge B. Claverie J. Courty S. Fremolle J. (1999). Microcephaly and macrocephaly in autism. J. Autism Dev. Disord. 29, 113–119 10.1023/A:1023036509476 10382131

[B109] Forster E. Jossin Y. Zhao S. Chai X. Frotscher M. Goffinet A. M. (2006). Recent progress in understanding the role of Reelin in radial neuronal migration, with specific emphasis on the dentate gyrus. Eur. J. Neurosci. 23, 901–909 10.1111/j.1460-9568.2006.04612.x 16519655

[B110] Friedman J. I. Vrijenhoek T. Markx S. Janssen I. M. van der Vliet W. A. Faas B. H. (2008). CNTNAP2 gene dosage variation is associated with schizophrenia and epilepsy. Mol. Psychiatry 13, 261–266 10.1038/sj.mp.4002049 17646849

[B111] Gambino F. Kneib M. Pavlowsky A. Skala H. Heitz S. Vitale N. (2009). IL1RAPL1 controls inhibitory networks during cerebellar development in mice. Eur. J. Neurosci. 30, 1476–1486 10.1111/j.1460-9568.2009.06975.x 19811529

[B112] Geschwind D. H. (2009). Advances in autism. Annu. Rev. Med. 60, 367–380 10.1146/annurev.med.60.053107.121225 19630577 PMC3645857

[B113] Gharani N. Benayed R. Mancuso V. Brzustowicz L. M. Millonig J. H. (2004). Association of the homeobox transcription factor, ENGRAILED 2, 3, with autism spectrum disorder. Mol. Psychiatry 9, 474–484 10.1038/sj.mp.4001498 15024396

[B114] Gillberg C. Wahlstrom J. (1985). Chromosome abnormalities in infantile autism and other childhood psychoses: a population study of 66 cases. Dev. Med. Child Neurol. 27, 293–304 10.1111/j.1469-8749.1985.tb04539.x 3160621

[B115] Gkogkas C. G. Khoutorsky A. Ran I. Rampakakis E. Nevarko T. Weatherill D. B. (2013). Autism-related deficits via dysregulated eIF4E-dependent translational control. Nature 493, 371–377 10.1038/nature11628 23172145 PMC4133997

[B116] Glessner J. T. Wang K. Cai G. Korvatska O. Kim C. E. Wood S. (2009). Autism genome-wide copy number variation reveals ubiquitin and neuronal genes. Nature 459, 569–573 10.1038/nature07953 19404257 PMC2925224

[B117] Goff D. C. Tsai G. Levitt J. Amico E. Manoach D. Schoenfeld D. A. (1999). A placebo-controlled trial of D-cycloserine added to conventional neuroleptics in patients with schizophrenia. Arch. Gen. Psychiatry 56, 21–27 10.1001/archpsyc.56.1.21 9892252

[B118] Gogolla N. Leblanc J. J. Quast K. B. Sudhof T. C. Fagiolini M. Hensch T. K. (2009). Common circuit defect of excitatory-inhibitory balance in mouse models of autism. J. Neurodev. Disord. 1, 172–181 10.1007/s11689-009-9023-x 20664807 PMC2906812

[B119] Goldstein S. Schwebach A. J. (2004). The comorbidity of pervasive developmental disorder and attention deficit hyperactivity disorder: results of a retrospective chart review. J. Autism Dev. Disord. 34, 329–339 10.1023/B:JADD.0000029554.46570.68 15264500

[B120] Goss A. M. Tian Y. Tsukiyama T. Cohen E. D. Zhou D. Lu M. M. (2009). Wnt2/2b and beta-catenin signaling are necessary and sufficient to specify lung progenitors in the foregut. Dev. Cell 17, 290–298 10.1016/j.devcel.2009.06.005 19686689 PMC2763331

[B121] Gould B. R. Zingg H. H. (2003). Mapping oxytocin receptor gene expression in the mouse brain and mammary gland using an oxytocin receptor-LacZ reporter mouse. Neuroscience 122, 155–167 10.1016/S0306-4522(03)00283-5 14596857

[B122] Govek E. E. Newey S. E. Akerman C. J. Cross J. R. Van der Veken L. Van Aelst L. (2004). The X-linked mental retardation protein oligophrenin-1 is required for dendritic spine morphogenesis. Nat. Neurosci. 7, 364–372 10.1038/nn1210 15034583

[B123] Grabrucker A. M. (2012). Environmental factors in autism. Front. Psychiatry 3:118 10.3389/fpsyt.2012.00118 23346059 PMC3548163

[B125] Greenberg M. E. Xu B. Lu B. Hempstead B. L. (2009). New insights in the biology of BDNF synthesis and release: implications in CNS function. J. Neurosci. 29, 12764–12767 19828787 10.1523/JNEUROSCI.3566-09.2009 PMC3091387

[B126] Gregory K. J. Dong E. N. Meiler J. Conn P. J. (2011). Allosteric modulation of metabotropic glutamate receptors: structural insights and therapeutic potential. Neuropharmacology 60, 66–81 10.1016/j.neuropharm.2010.07.007 20637216 PMC2981682

[B127] Grzadzinski R. Huerta M. Lord C. (2013). DSM-5 and autism spectrum disorders (ASDs): an opportunity for identifying ASD subtypes. Mol. Autism 4, 12 10.1186/2040-2392-4-12 23675638 PMC3671160

[B128] Guo X. Hamilton P. J. Reish N. J. Sweatt J. D. Miller C. A. Rumbaugh G. (2009). Reduced expression of the NMDA receptor-interacting protein SynGAP causes behavioral abnormalities that model symptoms of Schizophrenia. Neuropsychopharmacology 34, 1659–1672 10.1038/npp.2008.223 19145222 PMC3690772

[B129] Gutierrez H. Dolcet X. Tolcos M. Davies A. (2004). HGF regulates the development of cortical pyramidal dendrites. Development 131, 3717–3726 10.1242/dev.01209 15229174

[B130] Hamdan F. F. Daoud H. Piton A. Gauthier J. Dobrzeniecka S. Krebs M. O. (2011). De novo SYNGAP1 mutations in nonsyndromic intellectual disability and autism. Biol. Psychiatry 69, 898–901 10.1016/j.biopsych.2010.11.015 21237447

[B131] Han J. M. Sahin M. (2011). TSC1/TSC2 signaling in the CNS. FEBS Lett. 585, 973–980 10.1016/j.febslet.2011.02.001 21329690 PMC3070766

[B132] Han S. Tai C. Westenbroek R. E. Yu F. H. Cheah C. S. Potter G. B. (2012). Autistic-like behaviour in Scn1a+/- mice and rescue by enhanced GABA-mediated neurotransmission. Nature 489, 385–390 10.1038/nature11356 22914087 PMC3448848

[B133] Harrison D. E. Strong R. Sharp Z. D. Nelson J. F. Astle C. M. Flurkey K. (2009). Rapamycin fed late in life extends lifespan in genetically heterogeneous mice. Nature 460, 392–395 19587680 10.1038/nature08221 PMC2786175

[B134] Hava G. Vered L. Yael M. Mordechai H. Mahoud H. (2006). Alterations in behavior in adult offspring mice following maternal inflammation during pregnancy. Dev. Psychobiol. 48, 162–168 10.1002/dev.20116 16489598

[B135] Hazlett H. C. Poe M. Gerig G. Smith R. G. Provenzale J. Ross A. (2005). Magnetic resonance imaging and head circumference study of brain size in autism: birth through age 2 years. Arch. Gen. Psychiatry 62, 1366–1376 10.1001/archpsyc.62.12.1366 16330725

[B136] Herbert M. R. (2005). Large brains in autism: the challenge of pervasive abnormality. Neuroscientist 11, 417–440 10.1177/0091270005278866 16151044

[B137] Herbert M. R. Ziegler D. A. Deutsch C. K. O'Brien L. M. Lange N. Bakardjiev A., Jr. (2003). Dissociations of cerebral cortex, subcortical and cerebral white matter volumes in autistic boys. Brain 126, 1182–1192 10.1093/brain/awg110 12690057

[B138] Herbert M. R. Ziegler D. A. Makris N. Filipek P. A. Kemper T. L. Normandin J. J. (2004). Localization of white matter volume increase in autism and developmental language disorder. Ann. Neurol. 55, 530–540 10.1002/ana.20032 15048892

[B139] Hines R. M. Wu L. Hines D. J. Steenland H. Mansour S. Dahlhaus R. (2008). Synaptic imbalance, stereotypies, and impaired social interactions in mice with altered neuroligin 2 expression. J. Neurosci. 28, 6055–6067 10.1523/JNEUROSCI.0032-08.2008 18550748 PMC6670530

[B140] Hollander E. Bartz J. Chaplin W. Phillips A. Sumner J. Soorya L. (2007). Oxytocin increases retention of social cognition in autism. Biol. Psychiatry 61, 498–503 10.1016/j.biopsych.2006.05.030 16904652

[B141] Hollander E. Novotny S. Hanratty M. Yaffe R. DeCaria C. M. Aronowitz B. R. (2003). Oxytocin infusion reduces repetitive behaviors in adults with autistic and Asperger's disorders. Neuropsychopharmacology 28, 193–198 10.1038/sj.npp.1300021 12496956

[B142] Hood W. F. Compton R. P. Monahan J. B. (1989). D-cycloserine: a ligand for the N-methyl-D-aspartate coupled glycine receptor has partial agonist characteristics. Neurosci. Lett. 98, 91–95 10.1016/0304-3940(89)90379-0 2540460

[B143] Horev G. Ellegood J. Lerch J. P. Son Y. E. Muthuswamy L. Vogel H. (2011). Dosage-dependent phenotypes in models of 16p11.2 lesions found in autism. Proc. Natl. Acad. Sci. U.S.A. 108, 17076–17081 10.1073/pnas.1114042108 21969575 PMC3193230

[B144] Huang E. J. Reichardt L. F. (2001). Neurotrophins: roles in neuronal development and function. Annu. Rev. Neurosci. 24, 677–736 10.1146/annurev.neuro.24.1.677 11520916 PMC2758233

[B145] Hung A. Y. Futai K. Sala C. Valtschanoff J. G. Ryu J. Woodworth M. A. (2008). Smaller dendritic spines, weaker synaptic transmission, but enhanced spatial learning in mice lacking Shank1. J. Neurosci. 28, 1697–1708 10.1523/JNEUROSCI.3032-07.2008 18272690 PMC2633411

[B146] Husi H. Ward M. A. Choudhary J. S. Blackstock W. P. Grant S. G. (2000). Proteomic analysis of NMDA receptor-adhesion protein signaling complexes. Nat. Neurosci. 3, 661–669 10.1038/76615 10862698

[B147] Hwa V. Oh Y. Rosenfeld R. G. (1999). The insulin-like growth factor-binding protein (IGFBP) superfamily. Endocr. Rev. 20, 761–787 10.1210/er.20.6.761 10605625

[B148] Ingram J. L. Peckham S. M. Tisdale B. Rodier P. M. (2000a). Prenatal exposure of rats to valproic acid reproduces the cerebellar anomalies associated with autism. Neurotoxicol. Teratol. 22, 319–324 10840175 10.1016/s0892-0362(99)00083-5

[B149] Ingram J. L. Stodgell C. J. Hyman S. L. Figlewicz D. A. Weitkamp L. R. Rodier P. M. (2000b). Discovery of allelic variants of HOXA1 and HOXB1: genetic susceptibility to autism spectrum disorders. Teratology 62, 393–405 11091361 10.1002/1096-9926(200012)62:6<393::AID-TERA6>3.0.CO;2-V

[B150] International Molecular Genetic Study of Autism, C. (1998). A full genome screen for autism with evidence for linkage to a region on chromosome 7q. International Molecular Genetic Study of Autism Consortium. Hum. Mol. Genet. 7, 571–578 9546821 10.1093/hmg/7.3.571

[B151] International Molecular Genetic Study of Autism, C. (2001). A genomewide screen for autism: strong evidence for linkage to chromosomes 2q, 7q, and 16p. Am. J. Hum. Genet. 69, 570–581 11481586 10.1086/323264 PMC1235486

[B152] Iossifov I. Ronemus M. Levy D. Wang Z. Hakker I. Rosenbaum J. (2012). *De novo* gene disruptions in children on the autistic spectrum. Neuron 74, 285–299 10.1016/j.neuron.2012.04.009 22542183 PMC3619976

[B153] Itoh M. Ide S. Takashima S. Kudo S. Nomura Y. Segawa M. (2007). Methyl CpG-binding protein 2 (a mutation of which causes Rett syndrome) directly regulates insulin-like growth factor binding protein 3 in mouse and human brains. J. Neuropathol. Exp. Neurol. 66, 117–123 10.1097/nen.0b013e3180302078 17278996

[B154] Jacob S. Brune C. W. Carter C. S. Leventhal B. L. Lord C. Cook E. H. Jr. (2007). Association of the oxytocin receptor gene (OXTR) in Caucasian children and adolescents with autism. Neurosci. Lett. 417, 6–9 10.1016/j.neulet.2007.02.001 17383819 PMC2705963

[B155] Jamain S. Betancur C. Quach H. Philippe A. Fellous M. Giros B. (2002). Linkage and association of the glutamate receptor 6 gene with autism. Mol. Psychiatry. 7, 302–310 10.1038/sj.mp.4000979 11920157 PMC2547854

[B156] Jamain S. Quach H. Betancur C. Rastam M. Colineaux C. Gillberg I. C. (2003). Mutations of the X-linked genes encoding neuroligins NLGN3 and NLGN4 are associated with autism. Nat. Genet. 34, 27–29 10.1038/ng1136 12669065 PMC1925054

[B157] Jamain S. Radyushkin K. Hammerschmidt K. Granon S. Boretius S. Varoqueaux F. (2008). Reduced social interaction and ultrasonic communication in a mouse model of monogenic heritable autism. Proc. Natl. Acad. Sci. U.S.A. 105, 1710–1715 10.1073/pnas.0711555105 18227507 PMC2234209

[B158] Joyner A. L. (1996). Engrailed, Wnt and Pax genes regulate midbrain–hindbrain development. TIG 12, 15–20 10.1016/0168-9525(96)81383-7 8741855

[B159] Just M. A. Cherkassky V. L. Keller T. A. Kana R. K. Minshew N. J. (2007). Functional and anatomical cortical underconnectivity in autism: evidence from an FMRI study of an executive function task and corpus callosum morphometry. Cereb. cortex 17, 951–961 10.1093/cercor/bhl006 16772313 PMC4500121

[B160] Kalume F. Yu F. H. Westenbroek R. E. Scheuer T. Catterall W. A. (2007). Reduced sodium current in Purkinje neurons from Nav1.1 mutant mice: implications for ataxia in severe myoclonic epilepsy in infancy. J. Neurosci. 27, 11065–11074 17928448 10.1523/JNEUROSCI.2162-07.2007 PMC6672849

[B161] Kanner L. (1943). Autistic disturbances of affective contact. Nerv. Child 2, 217–250 4880460

[B162] Khelfaoui M. Denis C. van Galen E. de Bock F. Schmitt A. Houbron C. (2007). Loss of X-linked mental retardation gene oligophrenin1 in mice impairs spatial memory and leads to ventricular enlargement and dendritic spine immaturity. J. Neurosci. 27, 9439–9450 10.1523/JNEUROSCI.2029-07.2007 17728457 PMC6673114

[B163] Khurana V. Lu Y. Steinhilb M. L. Oldham S. Shulman J. M. Feany M. B. (2006). TOR-mediated cell-cycle activation causes neurodegeneration in a Drosophila tauopathy model. Curr. Biol. 16, 230–241 10.1016/j.cub.2005.12.042 16461276

[B164] Kim H. G. Kishikawa S. Higgins A. W. Seong I. S. Donovan D. J. Shen Y. (2008). Disruption of neurexin 1 associated with autism spectrum disorder. Am. J. Hum. Genet. 82, 199–207 10.1016/j.ajhg.2007.09.011 18179900 PMC2253961

[B165] Kim J. H. Liao D. Lau L. F. Huganir R. L. (1998). SynGAP: a synaptic RasGAP that associates with the PSD-95/SAP90 protein family. Neuron 20, 683–691 10.1016/S0896-6273(00)81008-9 9581761

[B166] King B. H. Wright D. M. Handen B. L. Sikich L. Zimmerman A. W. McMahon W., Jr. (2001). Double-blind, placebo-controlled study of amantadine hydrochloride in the treatment of children with autistic disorder. J. Am. Acad. Child Adolesc. Psychiatry 40, 658–665 10.1097/00004583-200106000-00010 11392343

[B167] Kinney G. G. O'Brien J. A. Lemaire W. Burno M. Bickel D. J. Clements M. K. (2005). A novel selective positive allosteric modulator of metabotropic glutamate receptor subtype 5 has *in vivo* activity and antipsychotic-like effects in rat behavioral models. J. Pharmacol. Exp. Ther. 313, 199–206 10.1124/jpet.104.079244 15608073

[B168] Kirsten T. B. Taricano M. Maiorka P. C. Palermo-Neto J. Bernardi M. M. (2010). Prenatal lipopolysaccharide reduces social behavior in male offspring. Neuroimmunomodulation 17, 240–251 10.1159/000290040 20203530

[B169] Kosaka H. Munesue T. Ishitobi M. Asano M. Omori M. Sato M. (2012). Long-term oxytocin administration improves social behaviors in a girl with autistic disorder. BMC Psychiatry 12:110 10.1186/1471-244X-12-110 22888794 PMC3466125

[B170] Krueger D. D. Bear M. F. (2011). Toward fulfilling the promise of molecular medicine in fragile X syndrome. Annu. Rev. Med. 62, 411–429 10.1146/annurev-med-061109-134644 21090964 PMC3100156

[B171] Kwon C. H. Luikart B. W. Powell C. M. Zhou J. Matheny S. A. Zhang W. (2006). Pten regulates neuronal arborization and social interaction in mice. Neuron 50, 377–388 10.1016/j.neuron.2006.03.023 16675393 PMC3902853

[B172] Lalonde R. Hayzoun K. Derer M. Mariani J. Strazielle C. (2004). Neurobehavioral evaluation of Reln-rl-orl mutant mice and correlations with cytochrome oxidase activity. Neurosci. Res. 49, 297–305 10.1016/j.neures.2004.03.012 15196778

[B173] Laumonnier F. Bonnet-Brilhault F. Gomot M. Blanc R. David A. Moizard M. P. (2004). X-linked mental retardation and autism are associated with a mutation in the NLGN4 gene, a member of the neuroligin family. Am. J. Hum. Genet. 74, 552–557 10.1086/382137 14963808 PMC1182268

[B174] Leblond C. S. Heinrich J. Delorme R. Proepper C. Betancur C. Huguet G. (2012). Genetic and functional analyses of SHANK2 mutations suggest a multiple hit model of autism spectrum disorders. PLoS Genet. 8:e1002521 10.1371/journal.pgen.1002521 22346768 PMC3276563

[B175] Lee H. J. Macbeth A. H. Pagani J. H. Young W. S. 3rd. (2009). Oxytocin: the great facilitator of life. Prog. Neurobiol. 88, 127–151 19482229 10.1016/j.pneurobio.2009.04.001 PMC2689929

[B176] Li X. Alafuzoff I. Soininen H. Winblad B. Pei J. J. (2005). Levels of mTOR and its downstream targets 4E-BP1, eEF2, and eEF2 kinase in relationships with tau in Alzheimer's disease brain. FEBS J. 272, 4211–4220 10.1111/j.1742-4658.2005.04833.x 16098202

[B177] Lijam N. Paylor R. McDonald M. P. Crawley J. N. Deng C. X. Herrup K. (1997). Social interaction and sensorimotor gating abnormalities in mice lacking Dvl1. Cell 90, 895–905 10.1016/S0092-8674(00)80354-2 9298901

[B178] Liu F. Grauer S. Kelley C. Navarra R. Graf R. Zhang G. (2008). ADX47273 [S-(4-fluoro-phenyl)-{3-[3-(4-fluoro-phenyl)-[1,2,4]-oxadiazol-5-yl]-piperidin-1- yl}-methanone]: a novel metabotropic glutamate receptor 5-selective positive allosteric modulator with preclinical antipsychotic-like and procognitive activities. J. Pharmacol. Exp. Ther. 327, 827–839 10.1124/jpet.108.136580 18753411

[B179] Liu X. Kawamura Y. Shimada T. Otowa T. Koishi S. Sugiyama T. (2010). Association of the oxytocin receptor (OXTR) gene polymorphisms with autism spectrum disorder (ASD) in the Japanese population. J. Hum. Genet. 55, 137–141 10.1038/jhg.2009.140 20094064

[B180] Logan C. Y. Nusse R. (2004). The Wnt signaling pathway in development and disease. Annu. Rev. Cell Dev. Biol. 20, 781–810 10.1146/annurev.cellbio.20.010403.113126 15473860

[B181] Long J. M. LaPorte P. Paylor R. Wynshaw-Boris A. (2004). Expanded characterization of the social interaction abnormalities in mice lacking Dvl1. Genes Brain Behav. 3, 51–62 10.1046/j.1601-183x.2003.00045.x 14960015

[B182] Lotter V. (1966). Epidemiology of autistic conditions in young children: I. Prevalence. Soc. Psychiatry 1, 124–137 10.1007/BF00584048

[B183] lwainsky H. (1988). Mode of action, biotransformation and pharmacokinetics of antituberculosis drugs in animals and man, in: antituberculosis Drugs. Handb. Exp. Pharmacol. 84, 399

[B184] Ma D. Salyakina D. Jaworski J. M. Konidari I. Whitehead P. L. Andersen A. N. (2009). A genome-wide association study of autism reveals a common novel risk locus at 5p14.1. Ann. Hum. Genet. 73, 263–273 10.1111/j.1469-1809.2009.00523.x 19456320 PMC2918410

[B185] Maecker H. T. Todd S. C. Levy S. (1997). The tetraspanin superfamily: molecular facilitators. FASEB J. 11, 428–442 9194523

[B186] Malagelada C. Ryu E. J. Biswas S. C. Jackson-Lewis V. Greene L. A. (2006). RTP801 is elevated in Parkinson brain substantia nigral neurons and mediates death in cellular models of Parkinson's disease by a mechanism involving mammalian target of rapamycin inactivation. J. Neurosci. 26, 9996–10005 10.1523/JNEUROSCI.3292-06.2006 17005863 PMC6674487

[B187] Malkova N. V. Yu C. Z. Hsiao E. Y. Moore M. J. Patterson P. H. (2012). Maternal immune activation yields offspring displaying mouse versions of the three core symptoms of autism. Brain Behav. Immun. 26, 607–616 10.1016/j.bbi.2012.01.011 22310922 PMC3322300

[B188] Mannaioni G. Marino M. J. Valenti O. Traynelis S. F. Conn P. J. (2001). Metabotropic glutamate receptors 1 and 5 differentially regulate CA1 pyramidal cell function. J. Neurosci. 21, 5925–5934 11487615 10.1523/JNEUROSCI.21-16-05925.2001 PMC6763150

[B189] Manor I. Eisenberg J. Tyano S. Sever Y. Cohen H. Ebstein R. P. (2001). Family-based association study of the serotonin transporter promoter region polymorphism (5-HTTLPR) in attention deficit hyperactivity disorder. Am. J. Med. Genet. 105, 91–95 11425009

[B190] Maranduba C. M. Sa Moreira E. Muller Orabona G. Pavanello R. C. Vianna-Morgante A. M. Passos-Bueno M. R. (2004). Does the P172H mutation at the TM4SF2 gene cause X-linked mental retardation? Am. J. Med. Genet. A 124A, 413–415 14735593 10.1002/ajmg.a.20401

[B191] Marchetto M. C. Carromeu C. Acab A. Yu D. Yeo G. W. Mu Y. (2010). A model for neural development and treatment of Rett syndrome using human induced pluripotent stem cells. Cell 143, 527–539 10.1016/j.cell.2010.10.016 21074045 PMC3003590

[B192] Martinowich K. Manji H. Lu B. (2007). New insights into BDNF function in depression and anxiety. Nat. Neurosci. 10, 1089–1093 10.1038/nn1971 17726474

[B193] Marui T. Funatogawa I. Koishi S. Yamamoto K. Matsumoto H. Hashimoto O. (2010). Association between autism and variants in the wingless-type MMTV integration site family member 2 (WNT2) gene. Int. J. Neuropsychopharmacol. 13, 443–449 10.1017/S1461145709990903 19895723

[B194] McPheeters M. L. Warren Z. Sathe N. Bruzek J. L. Krishnaswami S. Jerome R. N. (2011). A systematic review of medical treatments for children with autism spectrum disorders. Pediatrics 127, e1312–e1321 21464191 10.1542/peds.2011-0427

[B195] Mehta M. V. Gandal M. J. Siegel S. J. (2011). mGluR5-antagonist mediated reversal of elevated stereotyped, repetitive behaviors in the VPA model of autism. PLoS ONE 6:e26077 10.1371/journal.pone.0026077 22016815 PMC3189241

[B196] Minshew N. J. Williams D. L. (2007). The new neurobiology of autism: cortex, connectivity, and neuronal organization. Arch. Neurol. 64, 945–950 10.1001/archneur.64.7.945 17620483 PMC2597785

[B197] Miyazaki K. Narita N. Narita M. (2005). Maternal administration of thalidomide or valproic acid causes abnormal serotonergic neurons in the offspring: implication for pathogenesis of autism. Int. J. Dev. Neurosci. 23, 287–297 10.1016/j.ijdevneu.2004.05.004 15749253

[B198] Miyazaki K. Narita N. Sakuta R. Miyahara T. Naruse H. Okado N. (2004). Serum neurotrophin concentrations in autism and mental retardation: a pilot study. Brain Dev. 26, 292–295 10.1016/S0387-7604(03)00168-2 15165668

[B199] Monahan J. B. Handelmann G. E. Hood W. F. Cordi A. A. (1989). D-cycloserine, a positive modulator of the N-methyl-D-aspartate receptor, enhances performance of learning tasks in rats. Pharmacol. Biochem. Behav. 34, 649–653 10.1016/0091-3057(89)90571-6 2560209

[B200] Moreno-De-Luca A. Myers S. M. Challman T. D. Moreno-De-Luca D. Evans D. W. Ledbetter D. H. (2013). Developmental brain dysfunction: revival and expansion of old concepts based on new genetic evidence. Lancet Neurol. 12, 406–414 10.1016/S1474-4422(13)70011-5 23518333 PMC4013791

[B201] Moreno-Fuenmayor H. Borjas L. Arrieta A. Valera V. Socorro-Candanoza L. (1996). Plasma excitatory amino acids in autism. Invest. Clin. 37, 113–128 8718922

[B202] Moretti P. Bouwknecht J. A. Teague R. Paylor R. Zoghbi H. Y. (2005). Abnormalities of social interactions and home-cage behavior in a mouse model of Rett syndrome. Hum. Mol. Genet. 14, 205–220 10.1093/hmg/ddi016 15548546

[B203] Nadif Kasri N. Nakano-Kobayashi A. Malinow R. Li B. Van Aelst L. (2009). The Rho-linked mental retardation protein oligophrenin-1 controls synapse maturation and plasticity by stabilizing AMPA receptors. Genes Dev. 23, 1289–1302 10.1101/gad.1783809 19487570 PMC2701582

[B204] Naisbitt S. Kim E. Tu J. C. Xiao B. Sala C. Valtschanoff J. (1999). Shank, a novel family of postsynaptic density proteins that binds to the NMDA receptor/PSD-95/GKAP complex and cortactin. Neuron 23, 569–582 10.1016/S0896-6273(00)80809-0 10433268

[B205] Nakatani J. Tamada K. Hatanaka F. Ise S. Ohta H. Inoue K. (2009). Abnormal behavior in a chromosome-engineered mouse model for human 15q11-13 duplication seen in autism. Cell 137, 1235–1246 10.1016/j.cell.2009.04.024 19563756 PMC3710970

[B206] Narita N. Kato M. Tazoe M. Miyazaki K. Narita M. Okado N. (2002). Increased monoamine concentration in the brain and blood of fetal thalidomide- and valproic acid-exposed rat: putative animal models for autism. Pediatr. Res. 52, 576–579 12357053 10.1203/00006450-200210000-00018

[B207] Neale B. M. Kou Y. Liu L. Ma'Ayan A. Samocha K. E. Sabo A. (2012). Patterns and rates of exonic de novo mutations in autism spectrum disorders. Nature 485, 242–245 10.1038/nature11011 22495311 PMC3613847

[B208] Neves-Pereira M. Muller B. Massie D. Williams J. H. O'Brien P. C. Hughes A. (2009). Deregulation of EIF4E: a novel mechanism for autism. J. Med. Genet. 46, 759–765 10.1136/jmg.2009.066852 19556253

[B209] Niederhofer H. (2007). Glutamate antagonists seem to be slightly effective in psychopharmacologic treatment of autism. J. Clin. Psychopharmacol. 27, 317–318 10.1097/01.jcp.0000270082.30500.69 17502791

[B210] Nightingale S. (2012). Autism spectrum disorders. Nat. Rev. Drug Discov. 11, 745–746 10.1038/nrd3771 23000684

[B211] Niswender C. M. Conn P. J. (2010). Metabotropic glutamate receptors: physiology, pharmacology, and disease. Annu. Rev. Pharmacol. Toxicol. 50, 295–322 10.1146/annurev.pharmtox.011008.145533 20055706 PMC2904507

[B212] O'Kusky J. R. Ye P. D'Ercole A. J. (2000). Insulin-like growth factor-I promotes neurogenesis and synaptogenesis in the hippocampal dentate gyrus during postnatal development. J. Neurosci. 20, 8435–8442 11069951 10.1523/JNEUROSCI.20-22-08435.2000 PMC6773150

[B213] O'Roak B. J. Vives L. Fu W. Egertson J. D. Stanaway I. B. Phelps I. G. (2012a). Multiplex targeted sequencing identifies recurrently mutated genes in autism spectrum disorders. Science 338, 1619–1622 23160955 10.1126/science.1227764 PMC3528801

[B214] O'Roak B. J. Vives L. Girirajan S. Karakoc E. Krumm N. Coe B. P. (2012b). Sporadic autism exomes reveal a highly interconnected protein network of *de novo* mutations. Nature 485, 246–250 22495309 10.1038/nature10989 PMC3350576

[B215] Ogier M. Wang H. Hong E. Wang Q. Greenberg M. E. Katz D. M. (2007). Brain-derived neurotrophic factor expression and respiratory function improve after ampakine treatment in a mouse model of Rett syndrome. J. Neurosci. 27, 10912–10917 10.1523/JNEUROSCI.1869-07.2007 17913925 PMC6672830

[B216] Ozdinler P. H. Macklis J. D. (2006). IGF-I specifically enhances axon outgrowth of corticospinal motor neurons. Nat. Neurosci. 9, 1371–1381 10.1038/nn1789 17057708

[B217] Pan T. Rawal P. Wu Y. Xie W. Jankovic J. Le W. (2009). Rapamycin protects against rotenone-induced apoptosis through autophagy induction. Neuroscience 164, 541–551 10.1016/j.neuroscience.2009.08.014 19682553

[B218] Pandey U. B. Nie Z. Batlevi Y. McCray B. A. Ritson G. P. Nedelsky N. B. (2007). HDAC6 rescues neurodegeneration and provides an essential link between autophagy and the UPS. Nature 447, 859–863 10.1038/nature05853 17568747

[B219] Pavlowsky A. Gianfelice A. Pallotto M. Zanchi A. Vara H. Khelfaoui M. (2010). A postsynaptic signaling pathway that may account for the cognitive defect due to IL1RAPL1 mutation. Curr. Biol. 20, 103–115 10.1016/j.cub.2009.12.030 20096586

[B220] Peca J. Feliciano C. Ting J. T. Wang W. Wells M. F. Venkatraman T. N. (2011). Shank3 mutant mice display autistic-like behaviours and striatal dysfunction. Nature 472, 437–442 10.1038/nature09965 21423165 PMC3090611

[B221] Penagarikano O. Abrahams B. S. Herman E. I. Winden K. D. Gdalyahu A. Dong H. (2011). Absence of CNTNAP2 leads to epilepsy, neuronal migration abnormalities, and core autism-related deficits. Cell 147, 235–246 10.1016/j.cell.2011.08.040 21962519 PMC3390029

[B222] Persico A. M. D'Agruma L. Maiorano N. Totaro A. Militerni R. Bravaccio C. (2001). Reelin gene alleles and haplotypes as a factor predisposing to autistic disorder. Mol. Psychiatry. 6, 150–159 10.1038/sj.mp.4000850 11317216

[B223] Peterson S. L. (1992). 7-Chlorokynurenic acid antagonizes the anticonvulsant activity of D-cycloserine in maximal electroshock seizures. Epilepsy Res. 13, 73–81 10.1016/0920-1211(92)90009-I 1478199

[B224] Peterson S. L. Schwade N. D. (1993). The anticonvulsant activity of D-cycloserine is specific for tonic convulsions. Epilepsy Res. 15, 141–148 10.1016/0920-1211(93)90094-N 8370351

[B225] Phelan K. McDermid H. E. (2012). The 22q13.3 Deletion Syndrome (Phelan-McDermid Syndrome). Mol. Syndromol. 2, 186–201 22670140 10.1159/000334260 PMC3366702

[B226] Philippe A. Martinez M. Guilloud-Bataille M. Gillberg C. Rastam M. Sponheim E. (1999). Genome-wide scan for autism susceptibility genes. Paris Autism Research International Sibpair Study. Hum. Mol. Genet. 8, 805–812 10.1093/hmg/8.5.805 10196369

[B227] Pilarski R. Eng C. (2004). Will the real Cowden syndrome please stand up (again)? Expanding mutational and clinical spectra of the PTEN hamartoma tumour syndrome. J. Med. Genet. 41, 323–326 10.1136/jmg.2004.018036 15121767 PMC1735782

[B228] Pinto D. Pagnamenta A. T. Klei L. Anney R. Merico D. Regan R. (2010). Functional impact of global rare copy number variation in autism spectrum disorders. Nature 466, 368–372 20531469 10.1038/nature09146 PMC3021798

[B229] Pisani A. Gubellini P. Bonsi P. Conquet F. Picconi B. Centonze D. Bernardi G. Calabresi P. (2001). Metabotropic glutamate receptor 5 mediates the potentiation of N-methyl-D-aspartate responses in medium spiny striatal neurons. Neuroscience 106, 579–587 10.1016/S0306-4522(01)00297-4 11591458

[B230] Piton A. Gauthier J. Hamdan F. F. Lafreniere R. G. Yang Y. Henrion E. (2011). Systematic resequencing of X-chromosome synaptic genes in autism spectrum disorder and schizophrenia. Mol. Psychiatry 16, 867–880 10.1038/mp.2010.54 20479760 PMC3289139

[B231] Piton A. Michaud J. L. Peng H. Aradhya S. Gauthier J. Mottron L. (2008). Mutations in the calcium-related gene IL1RAPL1 are associated with autism. Hum. Mol. Genet. 17, 3965–3974 10.1093/hmg/ddn300 18801879

[B232] Poliak S. Gollan L. Martinez R. Custer A. Einheber S. Salzer J. L. Trimmer J. S. Shrager P. Peles E. (1999). Caspr2, a new member of the neurexin superfamily, is localized at the juxtaparanodes of myelinated axons and associates with K+ channels. Neuron 24, 1037–1047 10.1016/S0896-6273(00)81049-1 10624965

[B233] Posey D. J. Kem D. L. Swiezy N. B. Sweeten T. L. Wiegand R. E. McDougle C. J. (2004). A pilot study of D-cycloserine in subjects with autistic disorder. Am. J. Psychiatry 161, 2115–2117 10.1176/appi.ajp.161.11.2115 15514414

[B234] Powell E. M. Mars W. M. Levitt P. (2001). Hepatocyte growth factor/scatter factor is a motogen for interneurons migrating from the ventral to dorsal telencephalon. Neuron 30, 79–89 10.1016/S0896-6273(01)00264-1 11343646

[B235] Powell E. M. Muhlfriedel S. Bolz J. Levitt P. (2003). Differential regulation of thalamic and cortical axonal growth by hepatocyte growth factor/scatter factor. Dev. Neurosci. 25, 197–206 10.1159/000072268 12966217

[B236] Powers R. W. 3rd. Kaeberlein M. Caldwell S. D. Kennedy B. K. Fields S. (2006). Extension of chronological life span in yeast by decreased TOR pathway signaling. Genes Dev. 20, 174–184 10.1101/gad.1381406 16418483 PMC1356109

[B237] Purcell A. E. Jeon O. H. Zimmerman A. W. Blue M. E. Pevsner J. (2001). Postmortem brain abnormalities of the glutamate neurotransmitter system in autism. Neurology 57, 1618–1628 10.1212/WNL.57.9.1618 11706102

[B238] Quartermain D. Mower J. Rafferty M. F. Herting R. L. Lanthorn T. H. (1994). Acute but not chronic activation of the NMDA-coupled glycine receptor with D-cycloserine facilitates learning and retention. Eur. J. Pharmacol. 257, 7–12 10.1016/0014-2999(94)90687-4 8082709

[B239] Radyushkin K. Hammerschmidt K. Boretius S. Varoqueaux F. El-Kordi A. Ronnenberg A. (2009). Neuroligin-3-deficient mice: model of a monogenic heritable form of autism with an olfactory deficit. Genes Brain Behav. 8, 416–425 10.1111/j.1601-183X.2009.00487.x 19243448

[B240] Rammes G. Rupprecht R. Ferrari U. Zieglgansberger W. Parsons C. G. (2001). The N-methyl-D-aspartate receptor channel blockers memantine, MRZ 2/579 and other amino-alkyl-cyclohexanes antagonise 5-HT(3) receptor currents in cultured HEK-293 and N1E-115 cell systems in a non-competitive manner. Neurosci. Lett. 306, 81–84 10.1016/S0304-3940(01)01872-9 11403963

[B241] Rasmussen S. A. Friedman J. M. (2000). NF1 gene and neurofibromatosis 1. Am. J. Epidemiol. 151, 33–40 10.1093/oxfordjournals.aje.a010118 10625171

[B242] Ravikumar B. Vacher C. Berger Z. Davies J. E. Luo S. Oroz L. G. (2004). Inhibition of mTOR induces autophagy and reduces toxicity of polyglutamine expansions in fly and mouse models of Huntington disease. Nat. Genet. 36, 585–595 10.1038/ng1362 15146184

[B243] Richter J. D. Sonenberg N. (2005). Regulation of cap-dependent translation by eIF4E inhibitory proteins. Nature 433, 477–480 10.1038/nature03205 15690031

[B244] Riikonen R. Makkonen I. Vanhala R. Turpeinen U. Kuikka J. Kokki H. (2006). Cerebrospinal fluid insulin-like growth factors IGF-1 and IGF-2 in infantile autism. Dev. Med. Child Neurol. 48, 751–755 10.1017/S0012162206001605 16904022

[B245] Rinaldi T. Kulangara K. Antoniello K. Markram H. (2007). Elevated NMDA receptor levels and enhanced postsynaptic long-term potentiation induced by prenatal exposure to valproic acid. Proc. Natl. Acad. Sci. U.S.A. 104, 13501–13506 10.1073/pnas.0704391104 17675408 PMC1948920

[B246] Rodier P. M. Ingram J. L. Tisdale B. Croog V. J. (1997). Linking etiologies in humans and animal models: studies of autism. Reprod. Toxicol. 11, 417–422 10.1016/S0890-6238(97)80001-U 9100317

[B247] Rolf L. H. Haarmann F. Y. Grotemeyer K. H. Kehrer H. (1993). Serotonin and amino acid content in platelets of autistic children. Acta Psychiatr. Scand. 87, 312–316 10.1111/j.1600-0447.1993.tb03378.x 8517170

[B248] Rosenbrock H. Kramer G. Hobson S. Koros E. Grundl M. Grauert M. (2010). Functional interaction of metabotropic glutamate receptor 5 and NMDA-receptor by a metabotropic glutamate receptor 5 positive allosteric modulator. Eur. J. Pharmacol. 639, 40–46 10.1016/j.ejphar.2010.02.057 20371241

[B249] Rudolph U. Knoflach F. (2011). Beyond classical benzodiazepines: novel therapeutic potential of GABAA receptor subtypes. Nat. Rev. Drug Discov. 10, 685–697 10.1038/nrd3502 21799515 PMC3375401

[B250] Rutter M. (2000). Genetic studies of autism: from the 1970s into the millennium. J. Abnorm. Child Psychol. 28, 3–14 10.1023/A:1005113900068 10772346

[B251] Sadakata T. Washida M. Iwayama Y. Shoji S. Sato Y. Ohkura T. (2007). Autistic-like phenotypes in Cadps2-knockout mice and aberrant CADPS2 splicing in autistic patients. J. Clin. Invest. 117, 931–943 10.1172/JCI29031 17380209 PMC1821065

[B252] Salinger W. L. Ladrow P. Wheeler C. (2003). Behavioral phenotype of the reeler mutant mouse: effects of RELN gene dosage and social isolation. Behav. Neurosci. 117, 1257–1275 10.1037/0735-7044.117.6.1257 14674845

[B253] Sallmann S. Juttler E. Prinz S. Petersen N. Knopf U. Weiser T. (2000). Induction of interleukin-6 by depolarization of neurons. J. Neurosci. 20, 8637–8642 11102468 10.1523/JNEUROSCI.20-23-08637.2000 PMC6773078

[B254] Sanders S. J. Murtha M. T. Gupta A. R. Murdoch J. D. Raubeson M. J. Willsey A. J. (2012). *De novo* mutations revealed by whole-exome sequencing are strongly associated with autism. Nature 485, 237–241 10.1038/nature10945 22495306 PMC3667984

[B255] Santini E. Huynh T. N. MacAskill A. F. Carter A. G. Pierre P. Ruggero D. (2013). Exaggerated translation causes synaptic and behavioural aberrations associated with autism. Nature 493, 411–415 10.1038/nature11782 23263185 PMC3548017

[B256] Sato D. Lionel A. C. Leblond C. S. Prasad A. Pinto D. Walker S. (2012). SHANK1 Deletions in Males with Autism Spectrum Disorder. Am. J. Hum. Genet. 90, 879–887 10.1016/j.ajhg.2012.03.017 22503632 PMC3376495

[B257] Schmeisser M. J. Ey E. Wegener S. Bockmann J. Stempel A. V. Kuebler A. (2012). Autistic-like behaviours and hyperactivity in mice lacking ProSAP1/Shank2. Nature 486, 256–260 22699619 10.1038/nature11015

[B258] Schneider T. Przewlocki R. (2005). Behavioral alterations in rats prenatally exposed to valproic acid: animal model of autism. Neuropsychopharmacology 30, 80–89 10.1038/sj.npp.1300518 15238991

[B259] Schumann C. M. Amaral D. G. (2006). Stereological analysis of amygdala neuron number in autism. J. Neurosci. 26, 7674–7679 10.1523/JNEUROSCI.1285-06.2006 16855095 PMC6674270

[B260] Schumann C. M. Hamstra J. Goodlin-Jones B. L. Lotspeich L. J. Kwon H. Buonocore M. H. (2004). The amygdala is enlarged in children but not adolescents with autism; the hippocampus is enlarged at all ages. J. Neurosci. 24, 6392–6401 10.1523/JNEUROSCI.1297-04.2004 15254095 PMC6729537

[B261] Schuster G. M. Schmidt W. J. (1992). D-cycloserine reverses the working memory impairment of hippocampal-lesioned rats in a spatial learning task. Eur. J. Pharmacol. 224, 97–98 10.1016/0014-2999(92)94825-G 1451747

[B262] Seeman P. Caruso C. Lasaga M. (2008). Memantine agonist action at dopamine D2High receptors. Synapse 62, 149–153 10.1002/syn.20472 18000814

[B263] Sehgal S. N. Baker H. Vezina C. (1975). Rapamycin (AY-22,989), a new antifungal antibiotic. II. Fermentation, isolation and characterization. J. Antibiot. 28, 727–732 10.7164/antibiotics.28.727 1102509

[B264] Serajee F. J. Zhong H. Nabi R. Huq A. H. (2003). The metabotropic glutamate receptor 8 gene at 7q31: partial duplication and possible association with autism. J. Med. Genet. 40:e42 12676915 10.1136/jmg.40.4.e42 PMC1735437

[B265] Shahbazian M. Young J. Yuva-Paylor L. Spencer C. Antalffy B. Noebels J. (2002). Mice with truncated MeCP2 recapitulate many Rett syndrome features and display hyperacetylation of histone H3. Neuron 35, 243–254 10.1016/S0896-6273(02)00768-7 12160743

[B266] Sheng M. Kim E. (2000). The Shank family of scaffold proteins. J. Cell Sci. 113(Pt 11), 1851–1856 10806096 10.1242/jcs.113.11.1851

[B267] Shifman S. Johannesson M. Bronstein M. Chen S. X. Collier D. A. Craddock N. J. (2008). Genome-wide association identifies a common variant in the reelin gene that increases the risk of schizophrenia only in women. PLoS Genet. 4:e28 10.1371/journal.pgen.0040028 18282107 PMC2242812

[B268] Silverman J. L. Smith D. G. Rizzo S. J. Karras M. N. Turner S. M. Tolu S. S. (2012). Negative allosteric modulation of the mGluR5 receptor reduces repetitive behaviors and rescues social deficits in mouse models of autism. Sci. Transl. Med. 4:131ra51 10.1126/scitranslmed.3003501 22539775 PMC4904784

[B269] Silverman J. L. Tolu S. S. Barkan C. L. Crawley J. N. (2010a). Repetitive self-grooming behavior in the BTBR mouse model of autism is blocked by the mGluR5 antagonist MPEP. Neuropsychopharmacology 35, 976–989 20032969 10.1038/npp.2009.201 PMC2827881

[B271] Silverman J. L. Yang M. Lord C. Crawley J. N. (2010b). Behavioural phenotyping assays for mouse models of autism. Nat. Rev. Neurosci. 11, 490–502 20559336 10.1038/nrn2851 PMC3087436

[B270] Silverman J. L. Turner S. M. Barkan C. L. Tolu S. S. Saxena R. Hung A. Y. (2011). Sociability and motor functions in Shank1 mutant mice. Brain Res. 1380, 120–137 10.1016/j.brainres.2010.09.026 20868654 PMC3041833

[B272] Simonoff E. Pickles A. Charman T. Chandler S. Loucas T. Baird G. (2008). Psychiatric disorders in children with autism spectrum disorders: prevalence, comorbidity, and associated factors in a population-derived sample. J. Am. Acad. Child Adolesc. Psychiatry 47, 921–929 10.1097/CHI.0b013e318179964f 18645422

[B273] Singh K. Sun S. Vezina C. (1979). Rapamycin (AY-22,989), a new antifungal antibiotic. IV. Mechanism of action. J. Antibiot. 32, 630–645 10.7164/antibiotics.32.630 381274

[B274] Singh V. K. Warren R. Averett R. Ghaziuddin M. (1997). Circulating autoantibodies to neuronal and glial filament proteins in autism. Pediatr. Neurol. 17, 88–90 10.1016/S0887-8994(97)00045-3 9308986

[B275] Sirvio J. Ekonsalo T. Riekkinen P. Jr. Lahtinen H. Riekkinen P. Sr. (1992). D-cycloserine, a modulator of the N-methyl-D-aspartate receptor, improves spatial learning in rats treated with muscarinic antagonist. Neurosci. Lett. 146, 215–218 10.1016/0304-3940(92)90081-H 1491793

[B276] Skaar D. A. Shao Y. Haines J. L. Stenger J. E. Jaworski J. Martin E. R. (2005). Analysis of the RELN gene as a genetic risk factor for autism. Mol. Psychiatry 10, 563–571 10.1038/sj.mp.4001614 15558079

[B277] Smith S. E. Li J. Garbett K. Mirnics K. Patterson P. H. (2007). Maternal immune activation alters fetal brain development through interleukin-6. J. Neurosci. 27, 10695–10702 10.1523/JNEUROSCI.2178-07.2007 17913903 PMC2387067

[B278] Sousa I. Clark T. G. Toma C. Kobayashi K. Choma M. Holt R. (2009). MET and autism susceptibility: family and case-control studies. Eur. J. Hum. Genet. 17, 749–758 10.1038/ejhg.2008.215 19002214 PMC2685893

[B279] Sparks B. F. Friedman S. D. Shaw D. W. Aylward E. H. Echelard D. Artru A. A. (2002). Brain structural abnormalities in young children with autism spectrum disorder. Neurology 59, 184–192 10.1212/WNL.59.2.184 12136055

[B280] Spooren W. Lindemann L. Ghosh A. Santarelli L. (2012). Synapse dysfunction in autism: a molecular medicine approach to drug discovery in neurodevelopmental disorders. Trends Pharmacol. Sci. 33, 669–684 10.1016/j.tips.2012.09.004 23084458

[B281] Stark K. L. Xu B. Bagchi A. Lai W. S. Liu H. Hsu R. (2008). Altered brain microRNA biogenesis contributes to phenotypic deficits in a 22q11-deletion mouse model. Nat. Genet. 40, 751–760 10.1038/ng.138 18469815

[B282] Stefani M. R. Moghaddam B. (2010). Activation of type 5 metabotropic glutamate receptors attenuates deficits in cognitive flexibility induced by NMDA receptor blockade. Eur. J. Pharmacol. 639, 26–32 10.1016/j.ejphar.2010.01.028 20371234 PMC3359134

[B283] Stubbs E. G. (1976). Autistic children exhibit undetectable hemagglutination-inhibition antibody titers despite previous rubella vaccination. J. Autism Child. Schizophr. 6, 269–274 10.1007/BF01543467 1036494

[B284] Sudhof T. C. (2008). Neuroligins and neurexins link synaptic function to cognitive disease. Nature 455, 903–911 10.1038/nature07456 18923512 PMC2673233

[B285] Sun W. Funakoshi H. Nakamura T. (2002). Localization and functional role of hepatocyte growth factor (HGF) and its receptor c-met in the rat developing cerebral cortex. Brain Res. Mol. Brain Res. 103, 36–48 10.1016/S0169-328X(02)00168-7 12106690

[B286] Tabuchi K. Blundell J. Etherton M. R. Hammer R. E. Liu X. Powell C. M. (2007). A neuroligin-3 mutation implicated in autism increases inhibitory synaptic transmission in mice. Science 318, 71–76 10.1126/science.1146221 17823315 PMC3235367

[B287] Tain L. S. Mortiboys H. Tao R. N. Ziviani E. Bandmann O. Whitworth A. J. (2009). Rapamycin activation of 4E-BP prevents parkinsonian dopaminergic neuron loss. Nat. Neurosci. 12, 1129–1135 10.1038/nn.2372 19684592 PMC2745154

[B288] Takayanagi Y. Yoshida M. Bielsky I. F. Ross H. E. Kawamata M. Onaka T. (2005). Pervasive social deficits, but normal parturition, in oxytocin receptor-deficient mice. Proc. Natl. Acad. Sci. U.S.A. 102, 16096–16101 10.1073/pnas.0505312102 16249339 PMC1276060

[B289] Testa C. M. Standaert D. G. Landwehrmeyer G. B. Penney J. B. Jr. Young A. B. (1995). Differential expression of mGluR5 metabotropic glutamate receptor mRNA by rat striatal neurons. J. Comp. Neurol. 354, 241–252 10.1002/cne.903540207 7782501

[B290] Toma C. Hervas A. Torrico B. Balmana N. Salgado M. Maristany M. (2013). Analysis of two language-related genes in autism: a case-control association study of FOXP2 and CNTNAP2. Psychiatr. Genet. 23, 82–85 10.1097/YPG.0b013e32835d6fc6 23277129

[B291] Troca-Marin J. A. Alves-Sampaio A. Montesinos M. L. (2011). An increase in basal BDNF provokes hyperactivation of the Akt-mammalian target of rapamycin pathway and deregulation of local dendritic translation in a mouse model of Down's syndrome. J. Neurosci. 31, 9445–9455 10.1523/JNEUROSCI.0011-11.2011 21715609 PMC6623175

[B292] Troca-Marin J. A. Alves-Sampaio A. Montesinos M. L. (2012). Deregulated mTOR-mediated translation in intellectual disability. Prog. Neurobiol. 96, 268–282 10.1016/j.pneurobio.2012.01.005 22285767

[B293] Tropea D. Giacometti E. Wilson N. R. Beard C. McCurry C. Fu D. D. (2009). Partial reversal of Rett Syndrome-like symptoms in MeCP2 mutant mice. Proc. Natl. Acad. Sci. U.S.A. 106, 2029–2034 10.1073/pnas.0812394106 19208815 PMC2644158

[B294] Tropea D. Kreiman G. Lyckman A. Mukherjee S. Yu H. Horng S. (2006). Gene expression changes and molecular pathways mediating activity-dependent plasticity in visual cortex. Nat. Neurosci. 9, 660–668 10.1038/nn1689 16633343

[B295] Tsai P. T. Hull C. Chu Y. Greene-Colozzi E. Sadowski A. R. Leech J. M. (2012). Autistic-like behaviour and cerebellar dysfunction in Purkinje cell Tsc1 mutant mice. Nature. 488, 647–651 10.1038/nature11310 22763451 PMC3615424

[B296] Tu J. C. Xiao B. Naisbitt S. Yuan J. P. Petralia R. S. Brakeman P. (1999). Coupling of mGluR/Homer and PSD-95 complexes by the Shank family of postsynaptic density proteins. Neuron. 23, 583–592 10.1016/S0896-6273(00)80810-7 10433269

[B297] Uslaner J. M. Parmentier-Batteur S. Flick R. B. Surles N. O. Lam J. S. McNaughton C. H. (2009). Dose-dependent effect of CDPPB, the mGluR5 positive allosteric modulator, on recognition memory is associated with GluR1 and CREB phosphorylation in the prefrontal cortex and hippocampus. Neuropharmacology. 57, 531–538 10.1016/j.neuropharm.2009.07.022 19627999

[B298] Valnegri P. Khelfaoui M. Dorseuil O. Bassani S. Lagneaux C. Gianfelice A. (2011a). A circadian clock in hippocampus is regulated by interaction between oligophrenin-1 and Rev-erbalpha. Nat. Neurosci. 14, 1293–1301 21874017 10.1038/nn.2911

[B299] Valnegri P. Montrasio C. Brambilla D. Ko J. Passafaro M. Sala C. (2011b). The X-linked intellectual disability protein IL1RAPL1 regulates excitatory synapse formation by binding PTPdelta and RhoGAP2. Hum. Mol. Genet. 20, 4797–4809 21926414 10.1093/hmg/ddr418 PMC3221541

[B300] van Slegtenhorst M. de Hoogt R. Hermans C. Nellist M. Janssen B. Verhoef S. (1997). Identification of the tuberous sclerosis gene TSC1 on chromosome 9q34. Science 277, 805–808 10.1126/science.277.5327.805 9242607

[B301] Vargas D. L. Nascimbene C. Krishnan C. Zimmerman A. W. Pardo C. A. (2005). Neuroglial activation and neuroinflammation in the brain of patients with autism. Ann. Neurol. 57, 67–81 10.1002/ana.20315 15546155

[B302] Veenstra-Vanderweele J. Cook E. Jr. Lombroso P. J. (2003). Genetics of childhood disorders: XLVI. Autism, part 5: genetics of autism. J. Am. Acad. Child Adolesc. Psychiatry 42, 116–118 10.1097/00004583-200301000-00018 12500084

[B303] Vernes S. C. Newbury D. F. Abrahams B. S. Winchester L. Nicod J. Groszer M. (2008). A functional genetic link between distinct developmental language disorders. N. Engl. J. Med. 359, 2337–2345 10.1056/NEJMoa0802828 18987363 PMC2756409

[B304] Vezina C. Kudelski A. Sehgal S. N. (1975). Rapamycin (AY-22,989), a new antifungal antibiotic. I. Taxonomy of the producing streptomycete and isolation of the active principle. J. Antibiot. 28, 721–726 10.7164/antibiotics.28.721 1102508

[B305] Vincent J. B. Herbrick J. A. Gurling H. M. Bolton P. F. Roberts W. Scherer S. W. (2000). Identification of a novel gene on chromosome 7q31 that is interrupted by a translocation breakpoint in an autistic individual. Am. J. Hum. Genet. 67, 510–514 10.1086/303005 10889047 PMC1287197

[B306] Voineagu I. Wang X. Johnston P. Lowe J. K. Tian Y. Horvath S. (2011). Transcriptomic analysis of autistic brain reveals convergent molecular pathology. Nature 474, 380–384 10.1038/nature10110 21614001 PMC3607626

[B307] Wahlstrom J. Gillberg C. Gustavson K. H. Holmgren G. (1986). Infantile autism and the fragile X. A Swedish multicenter study. Am. J. Med. Genet. 23, 403–408 10.1002/ajmg.1320230132 3953658

[B308] Walsh T. McClellan J. M. McCarthy S. E. Addington A. M. Pierce S. B. Cooper G. M. (2008). Rare structural variants disrupt multiple genes in neurodevelopmental pathways in schizophrenia. Science 320, 539–543 10.1126/science.1155174 18369103

[B309] Wang K. Zhang H. Ma D. Bucan M. Glessner J. T. Abrahams B. S. (2009). Common genetic variants on 5p14.1 associate with autism spectrum disorders. Nature 459, 528–533 10.1038/nature07999 19404256 PMC2943511

[B310] Wang L. Jia M. Yue W. Tang F. Qu M. Ruan Y. (2008). Association of the ENGRAILED 2 (EN2) gene with autism in Chinese Han population. Am. J. Med. Genet. Part B Neuropsychiat. Genet. 147B, 434–438 17948901 10.1002/ajmg.b.30623

[B311] Wang X. McCoy P. A. Rodriguiz R. M. Pan Y. Je H. S. Roberts A. C. (2011). Synaptic dysfunction and abnormal behaviors in mice lacking major isoforms of Shank3. Hum. Mol. Genet. 20, 3093–3108 10.1093/hmg/ddr212 21558424 PMC3131048

[B312] Warburton P. Baird G. Chen W. Morris K. Jacobs B. W. Hodgson S. (2000). Support for linkage of autism and specific language impairment to 7q3 from two chromosome rearrangements involving band 7q31. Am. J. Med. Genet. 96, 228–234 10893502 10.1002/(sici)1096-8628(20000403)96:2<228::aid-ajmg20>3.0.co;2-g

[B313] Wassink T. H. Piven J. Vieland V. J. Huang J. Swiderski R. E. Pietila J. (2001). Evidence supporting WNT2 as an autism susceptibility gene. Am. J. Med. Genet. 105, 406–413 10.1002/ajmg.1401 11449391

[B314] Wei H. Dobkin C. Sheikh A. M. Malik M. Brown W. T. Li X. (2012). The therapeutic effect of memantine through the stimulation of synapse formation and dendritic spine maturation in autism and fragile X syndrome. PLoS ONE 7:e36981 10.1371/journal.pone.0036981 22615862 PMC3352866

[B315] Wei H. Zou H. Sheikh A. M. Malik M. Dobkin C. Brown W. T. (2011). IL-6 is increased in the cerebellum of autistic brain and alters neural cell adhesion, migration and synaptic formation. J. Neuroinflamm. 8, 52 10.1186/1742-2094-8-52 21595886 PMC3114764

[B316] Weiss L. A. Arking D. E. Gene Discovery Project of Johns Hopkins the Autism Consortium Daly M. J. Chakravarti A. (2009). A genome-wide linkage and association scan reveals novel loci for autism. Nature 461, 802–808 10.1038/nature08490 19812673 PMC2772655

[B317] Williams D. L. Jr. Lindsley C. W. (2005). Discovery of positive allosteric modulators of metabotropic glutamate receptor subtype 5 (mGluR5). Curr. Top. Med. Chem. 5, 825–846 10.2174/1568026054750290 16178729

[B318] Wilson C. Idziaszczyk S. Parry L. Guy C. Griffiths D. F. Lazda E. (2005). A mouse model of tuberous sclerosis 1 showing background specific early post-natal mortality and metastatic renal cell carcinoma. Hum. Mol. Genet. 14, 1839–1850 10.1093/hmg/ddi190 15888477

[B319] Wilson H. L. Wong A. C. Shaw S. R. Tse W. Y. Stapleton G. A. Phelan M. C. (2003). Molecular characterisation of the 22q13 deletion syndrome supports the role of haploinsufficiency of SHANK3/PROSAP2 in the major neurological symptoms. J. Med. Genet. 40, 575–584 10.1136/jmg.40.8.575 12920066 PMC1735560

[B320] Wlaz P. Baran H. Loscher W. (1994). Effect of the glycine/NMDA receptor partial agonist, D-cycloserine, on seizure threshold and some pharmacodynamic effects of MK-801 in mice. Eur. J. Pharmacol. 257, 217–225 10.1016/0014-2999(94)90132-5 8088343

[B321] Won H. Lee H.-R. Gee H. Y. Mah W. Kim J.-I. Lee J. (2012). Autistic-like social behaviour in Shank2-mutant mice improved by restoring NMDA receptor function. Nature 486, 261–265 10.1038/nature11208 22699620

[B322] Wu H. M. Tzeng N. S. Qian L. Wei S. J. Hu X. Chen S. H. (2009). Novel neuroprotective mechanisms of memantine: increase in neurotrophic factor release from astroglia and anti-inflammation by preventing microglial activation. Neuropsychopharmacology 34, 2344–2357 19536110 10.1038/npp.2009.64 PMC3655438

[B323] Wu S. Jia M. Ruan Y. Liu J. Guo Y. Shuang M. (2005). Positive association of the oxytocin receptor gene (OXTR) with autism in the Chinese Han population. Biol. Psychiatry 58, 74–77 10.1016/j.biopsych.2005.03.013 15992526

[B324] Yan Q. J. Rammal M. Tranfaglia M. Bauchwitz R. P. (2005). Suppression of two major Fragile X Syndrome mouse model phenotypes by the mGluR5 antagonist MPEP. Neuropharmacology 49, 1053–1066 10.1016/j.neuropharm.2005.06.004 16054174

[B325] Yizhar O. Fenno L. E. Prigge M. Schneider F. Davidson T. J. O'Shea D. J. (2011). Neocortical excitation/inhibition balance in information processing and social dysfunction. Nature 477, 171–178 10.1038/nature10360 21796121 PMC4155501

[B326] Yoshida T. Yasumura M. Uemura T. Lee S. J. Ra M. Taguchi R. (2011). IL-1 receptor accessory protein-like 1 associated with mental retardation and autism mediates synapse formation by trans-synaptic interaction with protein tyrosine phosphatase delta. J. Neurosci. 31, 13485–13499 10.1523/JNEUROSCI.2136-11.2011 21940441 PMC6623287

[B327] Young D. M. Schenk A. K. Yang S. B. Jan Y. N. Jan L. Y. (2010). Altered ultrasonic vocalizations in a tuberous sclerosis mouse model of autism. Proc. Natl. Acad. Sci. U.S.A. 107, 11074–11079 10.1073/pnas.1005620107 20534473 PMC2890736

[B328] Yrigollen C. M. Han S. S. Kochetkova A. Babitz T. Chang J. T. Volkmar F. R. (2008). Genes controlling affiliative behavior as candidate genes for autism. Biol. Psychiatry 63, 911–916 10.1016/j.biopsych.2007.11.015 18207134 PMC2386897

[B329] Yu F. H. Mantegazza M. Westenbroek R. E. Robbins C. A. Kalume F. Burton K. A. (2006). Reduced sodium current in GABAergic interneurons in a mouse model of severe myoclonic epilepsy in infancy. Nat. Neurosci. 9, 1142–1149 10.1038/nn1754 16921370

[B330] Zemni R. Bienvenu T. Vinet M. C. Sefiani A. Carrie A. Billuart P. (2000). A new gene involved in X-linked mental retardation identified by analysis of an X;2 balanced translocation. Nat. Genet. 24, 167–170 10.1038/72829 10655063

[B331] Zeng H. Shen E. H. Hohmann J. G. Oh S. W. Bernard A. Royall J. J. (2012). Large-scale cellular-resolution gene profiling in human neocortex reveals species-specific molecular signatures. Cell 149, 483–496 10.1016/j.cell.2012.02.052 22500809 PMC3328777

[B332] Zill P. Baghai T. C. Zwanzger P. Schule C. Eser D. Rupprecht R. (2004). SNP and haplotype analysis of a novel tryptophan hydroxylase isoform (TPH2) gene provide evidence for association with major depression. Mol. Psychiatry 9, 1030–1036 10.1038/sj.mp.4001525 15124006

